# Global increase in methane production under future warming of lake bottom waters

**DOI:** 10.1111/gcb.16298

**Published:** 2022-06-24

**Authors:** Joachim Jansen, Richard Iestyn Woolway, Benjamin M. Kraemer, Clément Albergel, David Bastviken, Gesa A. Weyhenmeyer, Rafael Marcé, Sapna Sharma, Sebastian Sobek, Lars J. Tranvik, Marjorie Perroud, Malgorzata Golub, Tadhg N. Moore, Love Råman Vinnå, Sofia La Fuente, Luke Grant, Don C. Pierson, Wim Thiery, Eleanor Jennings

**Affiliations:** ^1^ Department of Ecology and Genetics/Limnology Uppsala University Uppsala Sweden; ^2^ School of Ocean Sciences Bangor University Anglesey UK; ^3^ Ecosystem Research Department IGB Leibniz Institute of Freshwater Ecology and Inland Fisheries Berlin Germany; ^4^ European Space Agency Climate Office ECSAT, Harwell Campus Didcot Oxfordshire UK; ^5^ Department of Thematic Studies – Environmental Change Linköping University Linköping Sweden; ^6^ Catalan Institute for Water Research Girona Spain; ^7^ University of Girona Girona Spain; ^8^ Department of Biology York University Toronto Ontario Canada; ^9^ Institute for Environmental Sciences University of Geneva Genève Switzerland; ^10^ Centre for Freshwater and Environmental Studies Dundalk Institute of Technology Dundalk Ireland; ^11^ Department of Biological Sciences Virginia Tech Blacksburg Virginia USA; ^12^ Eawag, Swiss Federal Institute of Aquatic Science and Technology Surface Waters‐Research and Management Kastanienbaum Switzerland; ^13^ Department of Hydrology and Hydraulic Engineering Vrije Universiteit Brussel Brussels Belgium

**Keywords:** aquatic, climate change, greenhouse gases, limnology, methane, temperature, tropics

## Abstract

Lakes are significant emitters of methane to the atmosphere, and thus are important components of the global methane budget. Methane is typically produced in lake sediments, with the rate of methane production being strongly temperature dependent. Local and regional studies highlight the risk of increasing methane production under future climate change, but a global estimate is not currently available. Here, we project changes in global lake bottom temperatures and sediment methane production rates from 1901 to 2099. By the end of the 21st century, lake bottom temperatures are projected to increase globally, by an average of 0.86–2.60°C under Representative Concentration Pathways (RCPs) 2.6–8.5, with greater warming projected at lower latitudes. This future warming of bottom waters will likely result in an increase in methane production rates of 13%–40% by the end of the century, with many low‐latitude lakes experiencing an increase of up to 17 times the historical (1970–1999) global average under RCP 8.5. The projected increase in methane production will likely lead to higher emissions from lakes, although the exact magnitude of the emission increase requires more detailed regional studies.

## Graphical abstract

Projected changes in global lake bottom water temperatures (top) drive future increases in methanogenesis rates (bottom) under different climate warming scenarios (RCPs) by the end of the 21st century. Global lake temperature simulations of the ISIMIP2b Lake Sector (1901 to 2099), were combined with an Arrhenius‐type temperature function of methanogenesis derived from lake sediment incubations. While bottom water warming in northern lakes is muted by increased water column stratification, greater warming of lake bottom waters in the tropics, combined with increased temperature sensitivity of methanogenesis at higher temperatures suggest that tropical lakes will experience the largest increases in methane production.

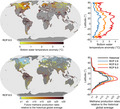

## 1 INTRODUCTION

Lakes, ponds, and reservoirs are globally significant emitters of the potent greenhouse gas methane (CH_4_) (Bastviken et al., 2011; Holgerson & Raymond, 2016; Rosentreter et al., 2021), and thus are important components of the global CH_4_ budget (Saunois et al., 2020, updated in Stavert et al., 2022). Lake CH_4_ is mostly produced by methanogenic Archaea in anoxic sediments (Bastviken et al., 2008) with the rates of CH_4_ production being strongly temperature dependent (Marotta et al., 2014; Segers, 1998; Zeikus & Winfrey, 1976). In turn, CH_4_ emissions exhibit an emergent temperature dependence that is robust across aquatic ecosystems and latitudes (Jansen, Thornton, Wik, et al., 2020; Wik et al., 2014; Yvon‐Durocher et al., 2014). Regional synthesis studies suggest that emissions of CH_4_ from lakes will increase within a warming world, potentially resulting in a positive feedback to climate change (Meredith et al., 2019; Walter et al., 2006; Wik et al., 2016). However, projected future changes in the temperature of lakes have primarily focused on surface waters (Woolway, Jennings, et al., 2021; Woolway & Maberly, 2020). Temperatures at the lake surface are rarely representative of the thermal environment at the sediment–water interface, and can even show contrasting patterns to historical climate change (Anderson et al., 2021; Kraemer et al., 2015; Pilla et al., 2020). No previous study has analyzed future changes in lake bottom water temperature at a global scale. Thus, while lake surface temperatures are projected to warm this century, there is a considerable knowledge gap of how future climatic warming will affect lake bottom temperatures and, subsequently, lake sediment CH_4_ production rates.

Here we quantified for the first time past and future changes in lake bottom water temperatures worldwide and directly assessed the global impact on CH_4_ production rates in lake sediments. We investigated the long‐term daily simulations of bottom water temperature from two independently developed and validated lake models (see Section 2; Figure S1), both driven with climate data from an ensemble of 20th‐ and 21st‐century climate projections, under three climate change scenarios (Representative Concentration Pathway [RCP]): RCP 2.6 (low‐emission scenario), 6.0 (medium–high emission scenario), and 8.5 (high‐emission scenario). Bottom temperatures from all lake‐climate model ensembles, which are available from the Inter‐Sectoral Impact Model Intercomparison Project phase 2b (ISIMIP2b) Lake Sector, were investigated from 1901 to 2099. Using the long‐term daily lake bottom temperatures, which we consider as a proxy for temperatures at the sediment–water interface, we then estimated historical and future changes in lake sediment CH_4_ production rates by applying a modified Arrhenius temperature function (Equation 1). The Arrhenius function has been widely used to express the exponential temperature sensitivity of metabolic processes, including CH_4_ production, across ecosystems (e.g., Gillooly et al., 2001; Kraemer et al., 2017; Yvon‐Durocher et al., 2014).

## 2 METHODS

### 2.1 Global lake simulations

Lake temperatures used in this study were simulated using two lake models, GOTM and SimStrat, which contributed to the ISIMIP2b lake sector (Golub et al., 2022). Specifically, the ISIMIP2b global lake simulations utilized GOTM v5.3 and SimStrat v1.4 with an added lake ice and snow module; SimStrat‐UoG (detailed model descriptions in Golub et al., 2022). The two models have been tested extensively in past studies including detailed validations across a spectrum of lake contexts. For example, SimStrat has previously been used to simulate lake temperature, as well as changes to stratification and mixing cycles in lakes, and has been shown to accurately reproduce temporal variations in bottom water temperature (Mesman et al., 2020; Perroud et al., 2009; Perroud & Goyette, 2010; Råman Vinnå et al., 2021). Most notably, the study of Peeters et al. (2002) demonstrated that the model is able to simulate hypolimnetic temperatures with a root mean square difference (RMSD) between simulated and observed temperatures of 0.34°C. Likewise, GOTM has been used to simulate the thermal environment of lakes across climatic regions, and has shown an excellent performance in simulating lake temperatures at depth in, for example, sub‐tropical (RMSD 0.63°C; Gal et al., 2020) and boreal lakes (RMSD 0.68°C; Ayala et al., 2020). Differences between the two models are negligible for our objective of simulating mean bottom water temperatures along a latitudinal gradient (Figure S1). To further confirm the robustness of our simulated bottom water temperatures, we validated model results against observational data from lakes (with any outliers removed using the Lake Analyzer software; Read et al., 2011) situated across latitudes and morphometric gradients (Figure S2). Although the models have some limitations (Text S1), we find good agreement between simulations and observations across the lakes tested.

Following the ISIMIP2b global lake sector protocol, each of these lake models were used to simulate lake temperatures at a 0.5°‐by‐0.5° grid resolution (Golub et al., 2022). For each 0.5° grid, the 1D models simulated daily temperature profiles in a cylindrical lake with the mean depth and surface area corresponding to the average of all known lakes within the grid. The ISIMIP2b lake sector simulation results have been applied successfully in global studies of thermal state changes in lakes associated with climate warming (Grant et al., 2021; Woolway, Sharma, et al., 2021). The locations, depths, and grid‐scale fractions of lakes within each 0.5° grid were determined by the Global Lake Data Base version 1 (GLDBv1), a rasterized dataset that integrates morphometry information from over 13,000 real lakes (Choulga et al., 2014; Kourzeneva, 2010; Subin et al., 2012). The 30‐arc second resolution of GLDBv1 sets the minimum lake surface area in each grid cell at approximately 1 km^2^. Lake information was aggregated from the original resolution of 30 arc seconds to a 0.5°‐by‐0.5° grid. Because larger lakes cover multiple lake pixels, the aggregated mean depth of the simulated lake in each grid cell represents an area‐weighted average. The ISIMIP2b lake sector simulations provide consistent projections of the thermal structure of over >17,000 representative lakes in a regularly gridded global data set. To drive each lake model, bias‐adjusted global climate model projections from the Coupled Model Intercomparison Project phase 5 (CMIP5) were used. Specifically, the lake models were driven by climate projections from GFDL‐ESM2M, HadGEM2‐ES, IPSL‐CM5A‐LR, and MIROC5 during the 20th‐ and 21st‐century under three greenhouse gas concentration scenarios: RCP 2.6, 6.0, and 8.5. The climate data used to drive each lake model included projections of air temperature at 2 m, wind speed at 10 m, downwelling surface solar and thermal radiation, and specific humidity. These data were used as inputs to the model after bias‐adjustment to the EWEMBI reference dataset (Frieler et al., 2017; Lange, 2019).

### 2.2 Lake stratification and mixing

Because the timing and frequency of lake mixing can have a strong impact on bottom water temperature (e.g., Bartosiewicz et al., 2019), we estimated the number of mixed days per year across the global distribution of studied lakes. A mixed day was estimated from the simulated lake temperature data and defined as the days in which a temperature‐driven density difference of 0.1 kg m^−3^ existed between surface and bottom waters. The number of mixing days per year was also compared between the historical and future projections, and we calculated both the absolute difference and the percent change throughout the study period.

### 2.3 Temperature dependence of methane production in lake sediments

We used an Arrhenius‐type temperature function (Yvon‐Durocher et al., 2014) to estimate the overall temperature sensitivity of methanogenesis from lake sediment incubations, and to project historical and future CH_4_ production rates in lake sediments:

(1) $$J\left(T\right)=J\left(T_{c}\right)e^{{E_{a}}^{\prime }\left(\frac{1}{k_{B}T_{c}}-\frac{1}{k_{B}T}\right)}$$

where *J* is the methanogenesis rate, *T* is the temperature in K, ${E_{a}}^{\prime }$ is the empirical activation energy in electron volts (eV), and *k*_B_ is the Boltzmann constant (8.62 × 10^−5^ eV K^−1^). ${E_{a}}^{\prime }$ quantifies the temperature sensitivity of the rate, and is equivalent to the slope of the natural logarithms of the centered rate ($J\left(T_{c}\right)-J\left(T\right)$) and temperature ($1/k_{B}T_{c}-1/k_{B}T$), where *J*(*T*_c_) is the rate at a midpoint temperature *T*_c_. We first estimated an overall temperature sensitivity of lake sediment CH_4_ production based on published incubation experiments from 30 unique sites in arctic, boreal, temperate, and tropical environments (Table S1). For one study (Marotta et al., 2014), tabular data were unavailable and measurements were extracted from figures using a plot digitizer (https://automeris.io/WebPlotDigitizer/). ${E_{a}}^{\prime }$ was estimated by fitting Equation (1) to the incubation measurements using a linear mixed‐effects model with site as a random effect (random intercepts and slopes). In turn, using Equation (1) and the ${E_{a}}^{\prime }$ value from the incubation experiments, we then computed CH_4_ production rates (*J*[*T*]) from each 0.5° grid cell temperature (*T*) as departures from an arbitrary historical global mean rate (*J*(*T*_c_) = 1) paired with a historical global mean bottom water temperature *T*_c_ from the lake models. By computing dimensionless rates relative to site‐specific and arbitrary global means, we avoided uncertainties associated with unit conversion between incubation studies and estimation of a global mean CH_4_ production rate. We used daily bottom water temperatures to compute CH_4_ production rates with Equation (1). We subsequently computed annual means of both the rates and the temperatures for each grid cell; therefore, the exponential temperature response of methanogenesis is fully resolved.

Based on the synthesis of published incubation experiments (Table S1), we estimated a mean ${E_{a}}^{\prime }$ of 0.96 eV. The use of a single ${E_{a}}^{\prime }$ estimate for our global simulations is consistent with the remarkably universal temperature sensitivity of methanogenesis across sediment incubations from global lakes, wetlands, and rice paddies (Yvon‐Durocher et al., 2014), which is also highly similar in lakes across climatic gradients (Marotta et al., 2014). However, while our estimates are based on the best available data and likely capture the overall temperature sensitivity of CH_4_ production, it should be taken into consideration that no truly global‐scale ${E_{a}}^{\prime }$ value exist, and that predictions about methane production in individual lakes require local validation. We performed a sensitivity analysis to test the robustness of our result against the assumption that ${E_{a}}^{\prime }$ varied randomly from site to site. In this case, the random site‐to‐site variation in ${E_{a}}^{\prime }$ was estimated from the distribution of observed values (95% confidence interval: 0.77–1.24 eV), which were then used in Equation (1) for the entire global lake simulation. That is, the global‐scale analysis described was repeated but using a random ${E_{a}}^{\prime }$ as opposed to a constant value.

## 3 RESULTS

### 3.1 Historical bottom water temperatures and CH_4_ production rates in lake sediments

**FIGURE 1:**
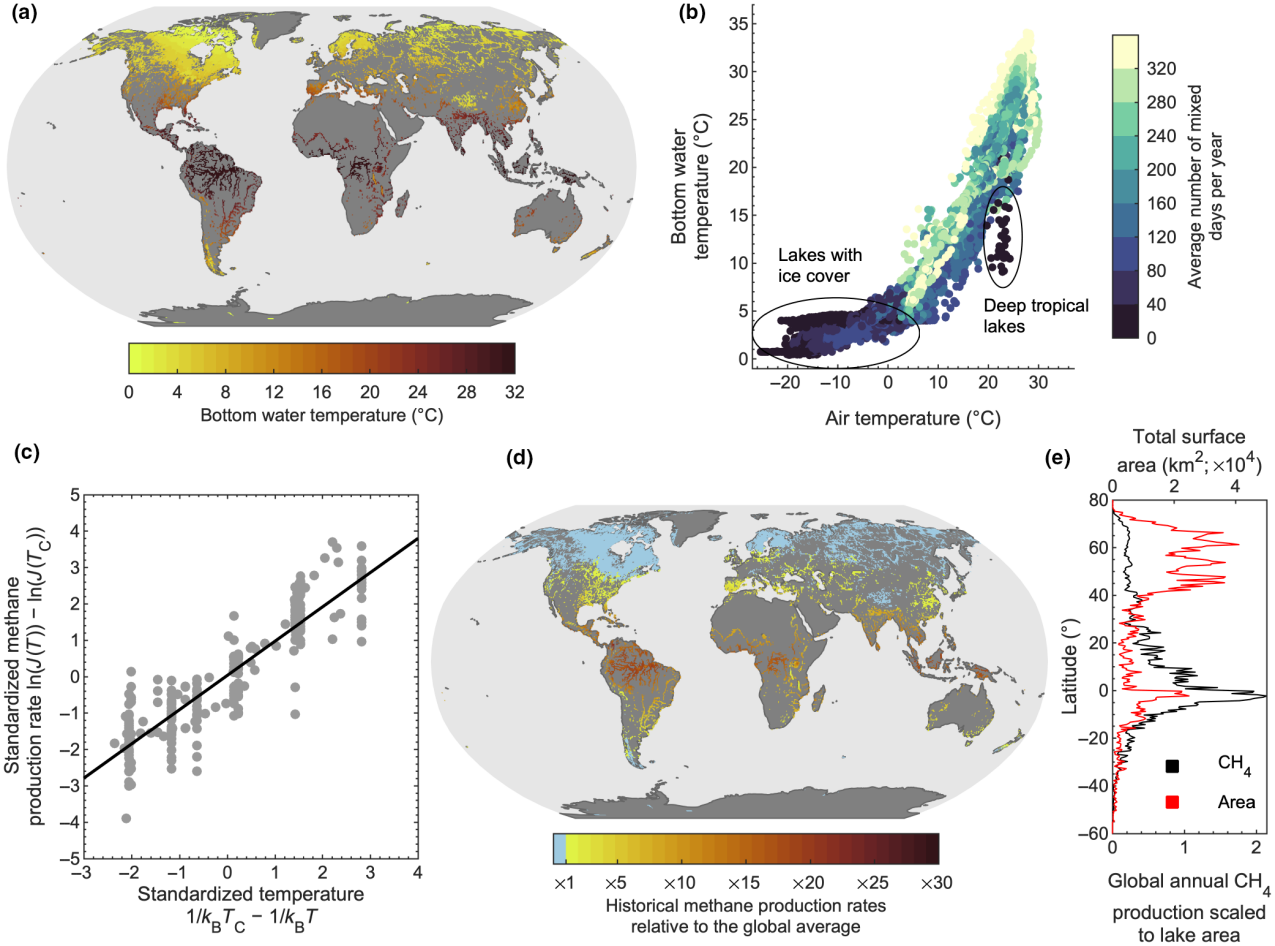
Historical simulations of annual mean bottom temperature and methane production rates in lake sediments. Shown are the historical, averaged over all years from 1970 to 1999 (a) spatial patterns in simulated annual (i.e., annual averages of the daily data) lake bottom temperatures and (b) the relationship between local (defined as the 0.5° grid cell in which a lake is situated) surface air and bottom water temperature in lakes, and the influence of the average number of mixed days per year (also see Figure S3). Also shown is (c), the temperature dependence of methane (CH_4_) production rates in lake sediments based on published incubation datasets from 30 unique sites (see Section 2); (d) the spatial patterns in the annual average CH_4_ production rates in lake sediments relative to the historical global average; and (e) the percentage of global lake sediment CH_4_ production as a function of latitude. All projections are based on the average simulations across the lake‐climate model ensemble. CH_4_ production rates are computed as annual averages of daily rates (i.e., using daily bottom water temperatures) to fully resolve the exponential temperature response of methanogenesis.

**FIGURE 2:**
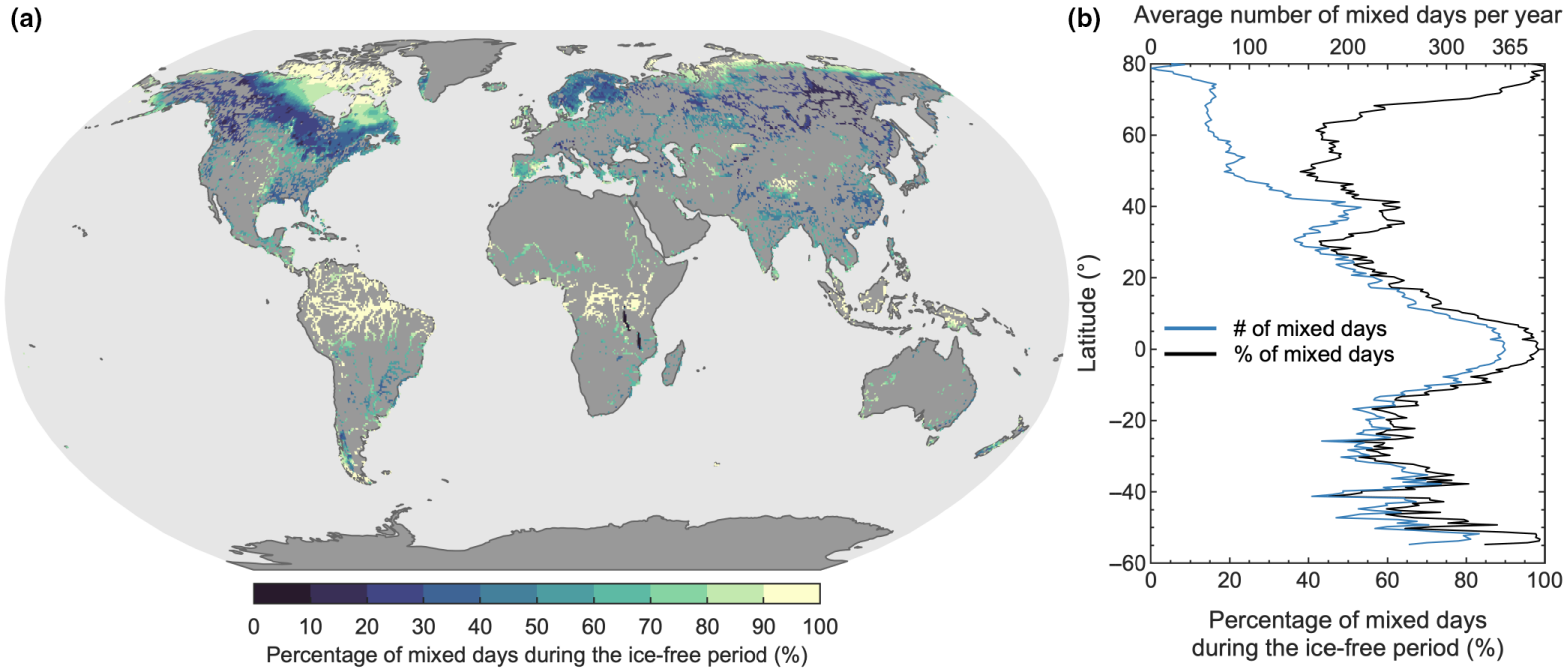
Global distribution in the frequency of lake mixing events each year. Shown for the historical period (1970–1999 average) are (a) the percentage of the ice‐free period when lakes are vertically mixed and (b) the latitudinal average (black line). Also shown in panel (b), is the absolute number of mixed days each year (blue line). All results represent the mean of the lake‐climate model ensemble.

Our multi‐model simulations suggest that lake bottom temperatures during the historical period (averaged here for all years from 1970 to 1999) vary along climatic gradients, with gradually increasing temperatures toward warmer geographic regions (Figure 1a). Some exceptions can be noted. The bottom temperatures of very deep, low‐latitude lakes, for example, tend to be colder compared to other lakes in the region (Figure 1b). One major reason for these exceptions is the influence of the lakes' stratification and mixing regime on the relation between lake bottom temperature and air temperature. The development of a density gradient severely limits turbulent heat exchange between bottom water and the surface layer in contact with the atmosphere. Therefore, on annual timescales, the temperature of air and bottom water can diverge in lakes that stratify either permanently or semi‐permanently (e.g., very deep low‐latitude lakes) (Figure 1b), or even for a sustained period of time during summer (e.g., medium to deep lakes at mid‐latitudes). In contrast, bottom water temperatures in lakes that mix regularly, such as the many shallow lakes located at both low and high latitudes (Figure 2), are influenced directly by climatic forcing, with bottom temperatures following closely the variability in local air temperature.

Given the overall latitudinal variation in lake bottom water temperature during the historical period, and the exponential temperature dependence of CH_4_ production in lake sediments, we found clear geographic patterns in simulated lake sediment CH_4_ production rates (Figure 1d). Here, for each simulated grid cell, we computed rates of methanogenesis relative to a historical global mean value using an Arrhenius‐type function (see Section 2). Our simulations suggest that lake sediment CH_4_ production rates are considerably greater at lower latitudes (Figure 1d). Most notably, based on our lake temperature simulations, we calculated that CH_4_ production rates in tropical (23.5°S–23.5°N) lake sediments are ∼12 times higher than the global average. In contrast, rates in the lake‐rich boreal region (50–70°N) are approximately half (∼0.52) the global average. Furthermore, our simulations suggest that 70% of the global CH_4_ produced in lake sediments during the historical period occurs in the tropics (Figure 1e). This is almost eight times greater than the total CH_4_ produced in boreal lakes (∼9% of global CH_4_ production), despite the total surface area covered by lakes in the boreal zone (41% of the total global lake surface area) being almost twice as large as in the tropics (23% of the total global lake surface area) (Figure S4).

### 3.2 Lake bottom temperatures and CH_4_ production rates under global warming

**FIGURE 3:**
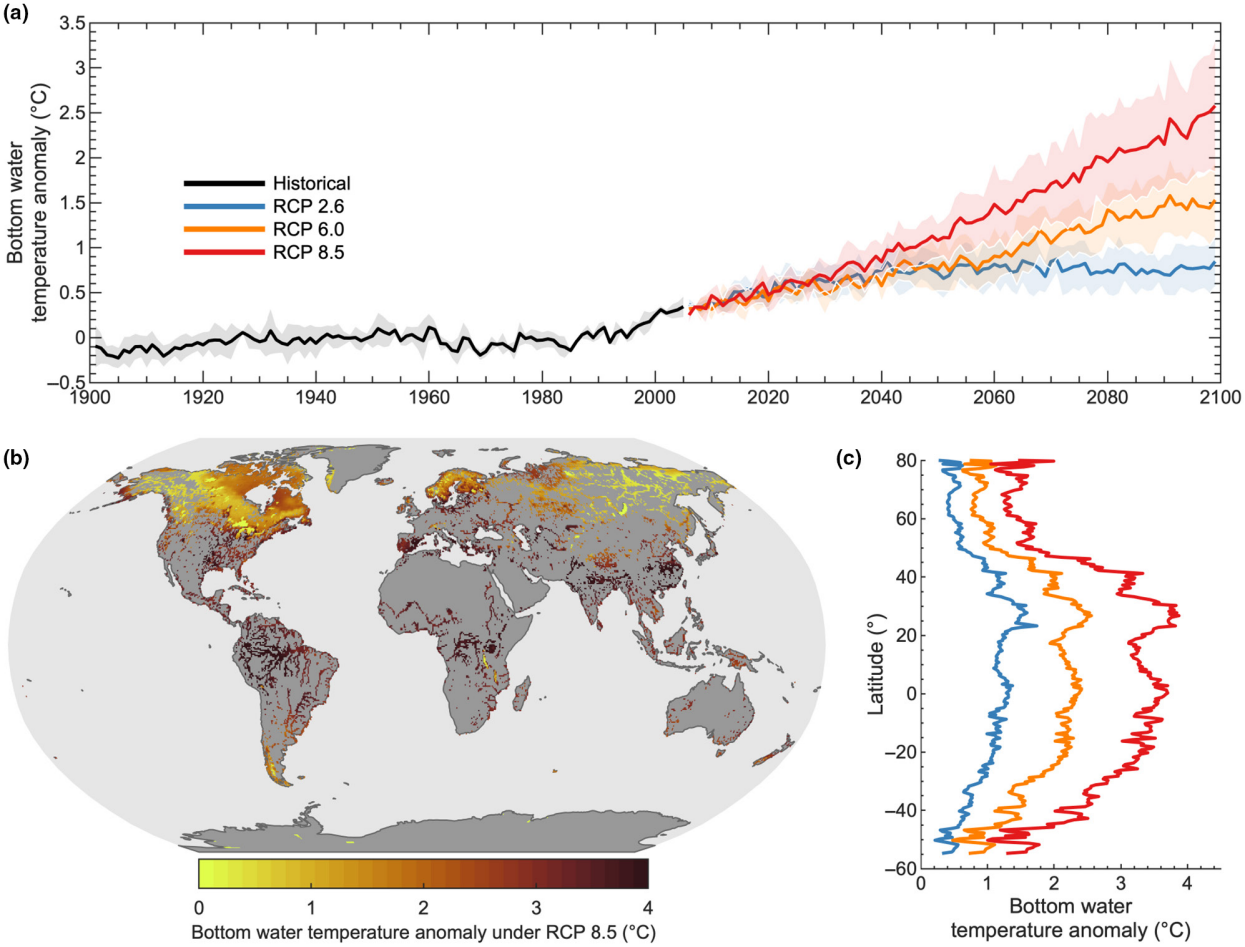
Historical and future projected change in global lake bottom water temperature. (a) Temporal changes in lake bottom water temperature from 1901 to 2099 under historical and future climate forcing. Future climate change scenarios (representative concentration pathway, RCP) include RCP 2.6 (low‐emission scenario), 6.0 (medium–high‐emission scenario), and 8.5 (high‐emission scenario). The thick lines show the global mean bottom temperature (i.e., across the lake‐climate model ensemble), and the shaded regions represent the standard deviation across the lake‐climate models. Anomalies are calculated relative to a 30‐year base period from 1970 to 1999. Also shown are (b) the spatial patterns in annual (i.e., annual averages of the daily data) lake bottom water temperature by 2070 to 2099 (i.e., averaged over all years) relative to the 1970 to 1999 base period average (described as anomalies) and (c) the latitudinal averages (0.5° bins) of future lake bottom temperature anomalies under RCP 2.6, 6.0, and 8.5. All results are based on the lake‐climate model ensemble.

During the 21st century, lake bottom water temperatures are projected to increase worldwide (Figure 2). Our multi‐model projections suggest that, by the end of the 21st century, global lake bottom temperatures will increase by an average of 0.9 ± 0.3°C, 1.6 ± 0.4°C, and 2.6 ± 0.7°C under RCP 2.6, 6.0, and 8.5, respectively, relative to historical conditions (1970–1999; quoted uncertainties represent the standard deviation from the lake‐climate model ensemble). Our projections also suggest that lake bottom temperatures will increase most rapidly at lower latitudes (Figure 3), which is contrary to the latitudinal variation in air temperature change suggested by the climate model projections (Figure S5).

**FIGURE 4:**
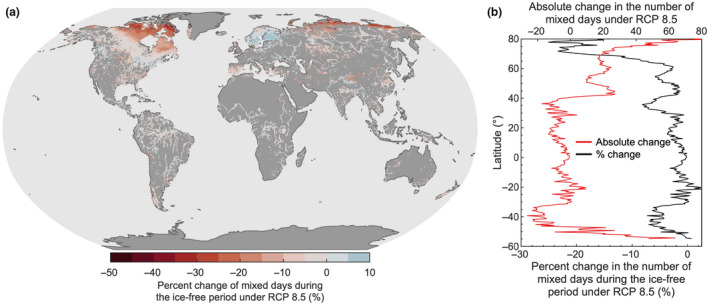
Future change in the frequency of lake mixing events. Shown are the differences between the number of mixed days between the historical (1970–1999 average) and future (2070–2099) periods. We show (a) the percentage change in the number of mixed days during the ice‐free period and (b) the latitudinal average (black line). Also shown in panel (b), is the absolute change in the number of mixed days each year (red line). All results represent the mean of the lake‐climate model ensemble.

The latitudinal variation in lake bottom temperature change during the 21st century is explained primarily by the global patterns of lake mixing, particularly the number of mixed days per year, as well as the magnitude of projected change in local air temperature. However, also interesting to consider is that many high‐latitude lakes will experience a decrease in the number of ice cover days this century (Grant et al., 2021), which likewise will result in an increase in the number of mixing days per year (Figure 4b). Even so, our projections suggest that the percentage of mixed days during the open‐water season will, in fact, decrease for high‐latitude lakes under the worst‐case climate change scenario (RCP 8.5) as they transition to a more stable stratification regime (Woolway & Merchant, 2019) and experience more stratified days during the open‐water season. Low‐latitude lakes are also projected to experience an increase in the number of stratified days this century, but the magnitude of change is considerably less than those projected at high latitude (Figure 4b). Ultimately, low‐latitude lakes will experience comparatively minimal change in the number of mixing days by the end of this century, that is, relative to high‐latitude lakes, as shown by both the absolute and percent change (Figure 4a,b). Therefore, low‐latitude lake bottom temperatures will follow more closely the long‐term change in air temperature this century.

The projected increase in global bottom water temperatures during the 21st century can be expected to result in an increase in lake sediment CH_4_ production rates which varies with latitude. The exponential nature of the Arrhenius function implies that the marginal rate change associated with a given temperature increment (e.g. 1°C) increases with temperature (Kraemer et al., 2017; Marotta et al., 2014). Lakes with historically high mean bottom temperatures could experience a considerable increase in CH_4_ production rates even if warming rates are relatively modest. In contrast, regions with historically low temperatures will require substantial warming of bottom waters to experience a comparatively moderate increase in CH_4_ production rates. Lakes that experience both historically high mean temperatures and substantial future warming, such as shallow lakes at low latitude (Figures 1 and 3), could experience an unprecedented increase in CH_4_ production rates during the current century.

**FIGURE 5:**
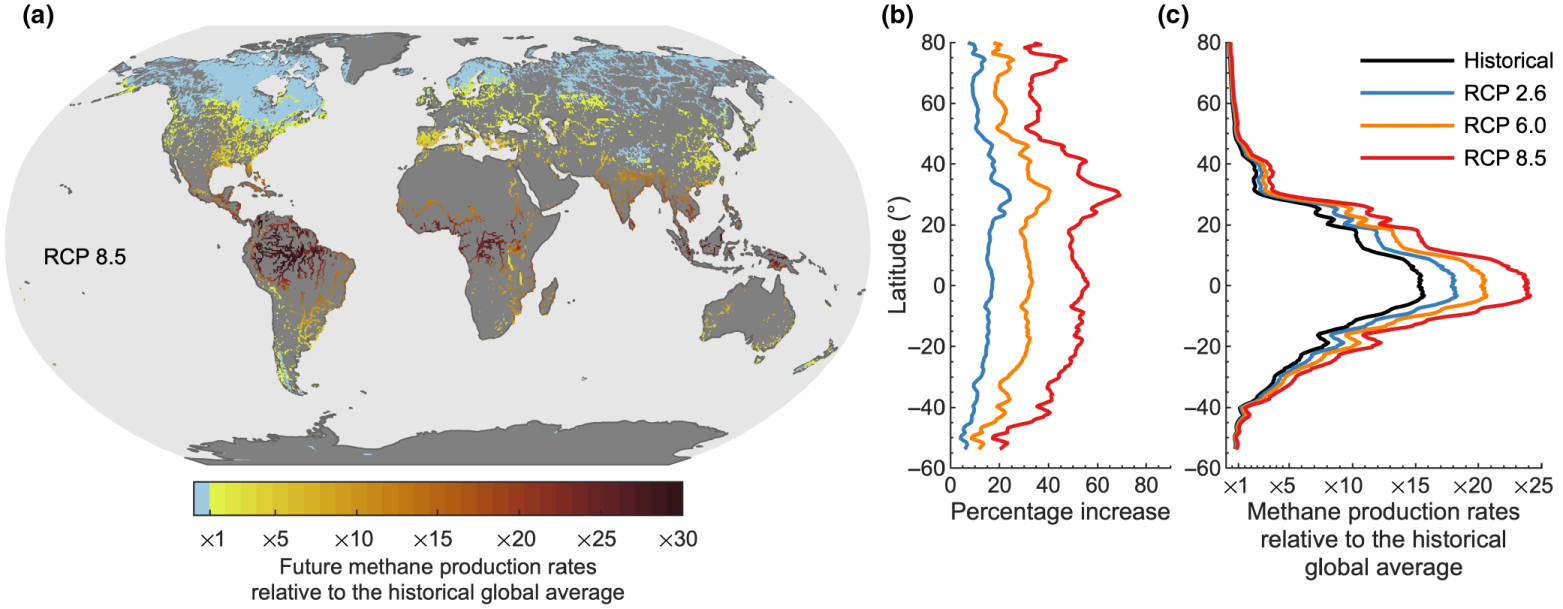
Future projections of methane production rates in lake sediments. (a) The spatial patterns in methane (CH_4_) production rates in lake sediments by the end of the 21st century (2070–2099) under representative concentration pathway (RCP) 8.5 (high‐emission scenario), relative to the historical (1970–1999) global average; (b) the percent increase in future CH_4_ production rates as a function of latitude under RCP 2.6 (low‐emission scenario), 6.0 (medium‐high‐emission scenario), and 8.5; and (c) the absolute change in lake sediment CH_4_ production rates during the historical period and by the end of the 21st century. All results are based on the lake‐climate model ensemble. In panels b and c, the averages are smoothed with a running mean across 2° latitudes.

Based on our daily future projections of bottom water temperatures, our simulations suggest that CH_4_ production rates will increase by the end of the 21st century relative to the historical period (Figure 5a). Specifically, our simulations show that global CH_4_ production rates will increase by 13%, 24%, and 40% of the historical global average under RCPs 2.6, 6.0, and 8.5, respectively. The percentage increase in methanogen productivity will be comparable across latitudes (Figure 5b), although with a higher percent increase in lakes situated at around 30°N. However, the absolute increase in CH_4_ production rates will be considerably higher in low‐latitude regions, due to both higher historical temperatures and greater future warming. Most notably, lake sediment CH_4_ production rates in tropical lakes will be 13, 15, and 17 times higher than the historical global average under RCP 2.6, 6.0, and RCP 8.5, respectively (Figure 5c). In some low‐latitude lakes, the rate of CH_4_ production was projected to be 30 times greater than the historical global average by the end of the 21st century under RCP 8.5 (Figure 5a). In contrast, future CH_4_ production rates at higher latitudes will, in many cases, remain below the historical global average production rates (Figure 5a).

### 3.3 Sensitivity of predicted CH_4_ production rates to variable temperature dependence

The thermal sensitivity of CH_4_ production in lake sediments is expressed here as the activation energy ${E_{a}}^{\prime }$ (Equation 1), with higher values representing a greater sensitivity to warming (Figure 1c). In the historical and future projections shown, we assumed a constant ${E_{a}}^{\prime }$ value for lakes worldwide, calculated from a global distribution of published lake sediment incubation experiments. While it is currently unfeasible to determine site‐specific ${E_{a}}^{\prime }$ for all lakes worldwide, which may also be unwarranted given that the temperature sensitivity of methanogenesis is remarkably similar across aquatic ecosystems (Yvon‐Durocher et al., 2014), we can test the sensitivity of our projections to global variations in ${E_{a}}^{\prime }$. For the sensitivity analysis, we investigated the influence on CH_4_ production rates due to a random global distribution of ${E_{a}}^{\prime }$, with site‐specific ${E_{a}}^{\prime }$ values chosen randomly from the distribution of observed values (see Section 2). Similar to when a constant ${E_{a}}^{\prime }$ was used, these results suggest higher CH_4_ production rates at low latitudes (Figure S6). These results further illustrate the dominant influence of the historical mean bottom temperature and the magnitude of 21st‐century warming, both of which are higher at low latitudes, on CH_4_ production rates.

## 4 DISCUSSION

### 4.1 Climatic drivers of lake bottom temperature

Our global simulations show that atmospheric warming has varying effects on the lake bottom temperature, depending, among other factors, on geographic location and lake morphometry. Specifically, (latitudinal) variations in stratification phenology shape the relation between annual mean air temperature and lake bottom water temperatures (Figure 1b). Lakes that stratify for a sustained period, such as those common at mid‐latitudes (Figure 2), often have cool bottom waters that are separated from the warmer layer above by a thermocline. Because this density gradient limits the downward penetration of atmospheric heat, bottom waters in these lakes receive the vast majority of their thermal energy during the period of homothermy (typically in winter/spring), with some additional heat gained during the stratified period via vertical diffusion (Wetzel, 2001). In lakes that are almost permanently stratified at annual timescales, such as the deep lakes south of the European Alps (e.g., Lake Garda) or those located at low latitudes (e.g., Lake Tanganyika), bottom water is, to a large extent, shielded from changes in air temperature. Instead, in these lakes, the temporal evolution of bottom temperature in recent decades has been characterized by a slow increase via the downward diffusion of heat (Ambrosetti & Barbanti, 1999; Verburg & Hecky, 2009). When these lakes eventually mix, for example, during exceptionally cold winters (Livingstone, 1997), bottom temperatures can cool abruptly.

In contrast, shallow lakes mix often and therefore the bottom water temperatures tend to be closely coupled to air temperatures on annual timescales (Figure 1b). However, also important for these lakes is the presence of seasonal ice cover, which influences the number of days in which bottom waters are exposed to surface heating. This means that the relationship between air temperature and bottom water temperature can differ between shallow lakes that freeze (e.g., high latitude/altitude lakes) and those that remain ice‐free (e.g., low‐altitude tropical lakes). Thus, while many high‐latitude lakes mix at regular intervals during the open‐water season and, in turn, their bottom temperatures are expected to closely follow the local air temperature, these lakes are ice‐covered for most of the year and thus are only exposed to atmospheric heating during a relatively short time period (Figure 2). Conversely, lakes without seasonal ice cover that mix regularly are exposed to surface heat exchange throughout the year, and bottom temperatures can closely follow the seasonal and inter‐annual variations in air temperature. Ultimately, the relationship between climate (e.g., air temperature) and bottom water temperature differs across lakes and is influenced by the seasonal evolution of stratification or the lack thereof.

Although our projections suggest an overall increase in global bottom water temperature, some lakes are projected to experience a decrease in bottom temperature during the 21st century at certain times of the year, in particular during the warmest months. Long‐term cooling of bottom waters in summer can be attributed to an increase in the strength and duration of thermal stratification (Bartosiewicz et al., 2019; Jankowski et al., 2006; Woolway, Sharma, et al., 2021) or even a shift in their mixing regime (Woolway & Merchant, 2019). Such changes will limit the duration in which bottom temperatures are exposed directly to atmospheric forcing. A decrease in historical bottom water temperature during summer has been observed recently by Pilla et al. (2020) and also previously by Kraemer et al. (2015). However, our model projections suggest that on annual timescales, for averages which are based on daily projections, lakes globally will experience an increase in lake bottom temperature during the 21st century, and the magnitude of bottom warming will be greater at lower latitudes.

### 4.2 Robustness of the temperature sensitivity of methanogenesis over space and time

In this study, we focused on the global implications of bottom temperature change on lake CH_4_ production rates, which was achieved using simulated bottom water temperatures within an Arrhenius‐type function. This is a valid approach, given that the activation energy of CH_4_ production in aquatic sediments is very similar to that of pure methanogenesis and robust across a variety of diverse ecosystem types (lake sediment, wetland soils, rice paddy sediment; Yvon‐Durocher et al., 2014). At a biochemical level, thermal optima of enzyme activity ensure that metabolic rates do not increase with temperature indefinitely, as the empirical Arrhenius function implies (DeLong et al., 2017), which is why it is important that we apply Equation (1) only within the temperature range of the incubation experiments where Equation (1) fits the data well. Changes in lake biogeochemistry and microbial communities that influence methanogenesis rates are often linked to shifts in temperature (Tveit et al., 2015). For example, decreasing oxygen penetration depth in the sediment at higher temperatures (Sobek et al., 2017) and increasing primary production in warmer climates (Yvon‐Durocher et al., 2017) may increase the bioavailability of fresh organic matter which is associated with enhanced CH_4_ production (Grasset et al., 2018; Whiting & Chanton, 1993), but this production will also be strongly temperature dependent. Bottom water warming will likely continue to be a main driver of CH_4_ production increases in the 21st century.

A possible caveat to our future projections is that the temperature dependency of methanogenesis may change over time. The ${E_{a}}^{\prime }$ values considered here are derived from relatively short‐term incubation measurements where, in brief, the temperature sensitivity of CH_4_ production is determined by raising the temperature of the sediment, while the amount of organic matter remains approximately the same, the redox regime is maintained, and the microbial community has little time to evolve. In natural systems, the rate of methanogenesis may be constrained by the amount and quality of organic matter (a function of internal primary production as well as external inputs of terrestrial organic carbon) or enhanced by deoxygenation of the surface sediment (a function of lake stratification and microbial oxygen consumption) and the long‐term adaptation of microbial communities (Blake et al., 2015; Borrel et al., 2011). Laboratory and mesocosm experiments have demonstrated synergistic effects of eutrophication and warming on methane production, effectively increasing the ${E_{a}}^{\prime }$ value (Beaulieu et al., 2019; Davidson et al., 2018; Tveit et al., 2015). A decade‐long warming experiment in artificial ponds indicated that methanogenic community shifts associated with warming can result in an increased ${E_{a}}^{\prime }$ value if paired with increased substrate abundance (from 0.7 to 1.1–1.4 eV) (Zhu et al., 2020). These studies suggest that over longer timescales, a combination of environmental changes may be expected to enhance both the lake methane production rate and its temperature sensitivity.

However, while controlled experiments suggest the possibility of future increases in the temperature sensitivity of methanogenesis, it is unclear whether similar changes in ${E_{a}}^{\prime }$ will take place in natural systems and at a global scale. For example, even under RCP 8.5, the temperature increases predicted for a majority of lakes by the end of the 21st century in our study (Figure 3) are less extreme than those in artificial warming experiments (up to 5°C) (Yvon‐Durocher et al., 2017; Zhu et al., 2020). Moreover, the sediment incubation experiments from the 30 different sites (Table S1) represent tropical, temperate, and boreal lake ecosystems, with distinct warming histories, organic matter properties and supply rates, stratification phenology and likely diverse microbial communities, yet they consistently show a highly similar temperature response of CH_4_ production. These temperature functions of CH_4_ production are consistent with those of CH_4_ emissions from natural systems (Yvon‐Durocher et al., 2014), which have already been subjected to warming over the past century. This suggests that ${E_{a}}^{\prime }$ values are relatively uniform geographically as well as consistent over time. In addition, our sensitivity analysis indicates that even with substantial, random variations in ${E_{a}}^{\prime }$, global geographic patterns in lake CH_4_ production changes are conserved (Figure S6).

Even so, the multitude of environmental changes that global lakes are subject to in addition to warming, such as deoxygenation (Jane et al., 2021) and browning (Solomon et al., 2015), are likely to affect CH_4_ production rates in the future. While we quantify the effect of one very important driver (temperature) of CH_4_ production rates, for which there is good fundamental understanding that allows modelling at the global scale, we also recognize the need to include the effects of synergetic and modulating processes which as yet are not well constrained enough to allow global application.

### 4.3 Do geographic patterns of methanogenesis rates depend primarily on temperature?

In this study, we have modelled the expected geographic and latitudinal variation of lake methanogenesis rates on a global scale based on spatiotemporal patterns in the lake bottom temperature. In reality, between‐lake differences in methane production are attributed to a variety of environmental factors besides temperature, such as lake productivity and the quality and quantity of organic substrate available to methanogens (Beaulieu et al., 2019; Emilson et al., 2018; Wik et al., 2018). Incubation experiments could help disentangle these drivers by comparing rates between sites at similar temperatures. Unfortunately, the different experimental designs of sediment incubation studies (Table S1) prohibit direct comparisons that would help to verify the modelled geographic patterns. One exception is the study of Marotta et al. (2014), which directly compares sediments from boreal and tropical lakes in a single experiment. We estimated the mean annual methanogenesis rate for each of the 17 sites in Marotta et al. with Equation (1), using modelled daily historical lake bottom temperatures at each site's corresponding 0.5°‐by‐0.5° grid and setting ${E_{a}}^{\prime }$ = 0.96 eV and *T*_C_ to the local annual mean temperature rather than the global mean. For each site, *J*(*T*_C_) was estimated via log‐linear regression of the rate versus temperature, and daily rates (*J*(*T*)) were averaged to annual historical mean rates. We finally compared the ratio of empirical tropical and boreal rate means to the ratio of modelled relative rates (Figure 1b) at the same grid cells. 95% confidence intervals of the ratios were computed following the method of Fieller (1954), using bootstrapped standard deviations (Table S3).

The incubation data support our assumption that temperature is the main driver of latitudinal gradients in CH_4_ production rates, but also point to potential biases. Given the historical lake bottom temperatures at each site, tropical lake sediments in the incubation study of Marotta et al. (2014) would be about 9.5 (95% CI 2.7–41.7) times more productive compared to boreal lake sediments on an annual basis. The wide confidence intervals of the ratio reflect the inter‐lake variability of methanogenesis rates, rather than of temperature, within each biome. Our simulation predicts a factor of 19.3 (95% CI 17.3–21.8), significantly higher but within the confidence bands of the empirical estimate. In comparison, at equivalent incubation temperatures and sediment volumes, boreal lake sediments tend to produce about 2.2 (95% CI 1.2–3.2) times more CH_4_ than tropical lake sediments. Thus, even though temperature effects appear to dominate, the modelled geographic distribution of methanogenesis rates could be biased by underprediction in boreal regions compared to the tropics. Even so, a factor 2.2 increase of historical methanogenesis rates in boreal lakes would not significantly alter the historical or projected future latitudinal gradients estimated from lake bottom temperature alone (Figure 1d).

### 4.4 Implications of increasing methane production rates for future methane emissions

The increase in CH_4_ production rates, as we have projected by the end of the 21st century, will likely result in higher CH_4_ emissions from lakes. This assessment appears to be supported by the robust “emergent” ecosystem‐level temperature sensitivity of global lake methane emissions (Aben et al., 2017; Sanches et al., 2019; Wik et al., 2014). However, a direct coupling of CH_4_ production and emission rates is confounded by temperature effects other than microbial metabolism, such as thermal stratification of the water column, and the solubility of CH_4_, which affects the dominant emission pathway (Jansen, Thornton, Cortés, et al., 2020; Jansen, Thornton, Wik, et al., 2020). Transfer of CH_4_ from lake sediments to the atmosphere can occur via (i) the formation and release of bubbles that quickly rise from the sediment through the water column to the atmosphere (ebullition) and (ii) diffusive transport of dissolved CH_4_ from the sediment to the water column and thereafter via turbulence‐driven transport to the air–water interface (diffusion). The latter pathway however also offers an opportunity for microbial oxidation of CH_4_ to CO_2_ at the sediment–water interface and in the water column (Duc et al., 2010; Lofton et al., 2014; Segers, 1998), which reduces the overall warming potential of the released carbon gas. While CH_4_ oxidation is expected to scale with CH_4_ production to an extent due to the former's dependence on substrate concentration (Duc et al., 2010), some studies suggest that warming disproportionally increases methanogenesis over methanotrophy (Bastviken 2009; Zhu et al., 2020). Furthermore, as production rates increase, so does the likelihood of CH_4_ concentrations in the sediment porewater that exceed supersaturation, leading to the growth of gas bubbles. Once this threshold is passed, any additional produced CH_4_ enters the gas phase and may leave the sediment via ebullition rather than diffusion. A shift in the predominant emission pathway may exacerbate emission rates relative to our projections of CH_4_ production rates because ebullition to a large extent bypasses methane oxidizers, and because of a higher ${E_{a}}^{\prime }$‐value of ebullition (DelSontro et al., 2016; Jansen, Thornton, Wik, et al., 2020) compared to sediment production.

Given the limited information about the coupling between CH_4_ production and emissions, it is at present not possible to translate our future CH_4_ production rates to emission scenarios. Therefore, in this first global assessment, we only address the fundamental question of how much CH_4_ production rates are likely to proportionately increase as a direct result of future warming of lake bottom waters. Even so, our simulated CH_4_ production rates do suggest the potential for subsequent increased atmospheric emissions during the current century. This notion is consistent with regional projections of lake CH_4_ emissions (Tan & Zhuang, 2015; Walter et al., 2007; Wik et al., 2016). Regional climate feedbacks not captured in our modeling efforts may further increase emission rates in the near future. Tan and Zhuang (2015) coupled a process‐based biogeochemistry model (Tan et al., 2015) to an arctic landscape evolution model and CMIP5 global warming scenarios to estimate pan‐Arctic (>60°N) lake CH_4_ emissions during the 21st century. Their simulations show that the majority (68%) of the increase in lake methane emissions can be attributed to direct and indirect temperature effects, which include an increasing supply of labile Pleistocene‐age carbon substrate to methanogens via thawing of sub‐surface permafrost beneath thermokarst lakes (Walter et al., 2007), and the remainder to the formation of new lake surface area due to terrestrial permafrost thaw (Walter Anthony et al., 2016). Importantly, these processes can generate key CH_4_ emission hotspots in permafrost‐rich regions that are only partially reflected in our global assessment of methanogenesis rates. Improving future predictions of lake CH_4_ emissions requires models that account not only for the direct temperature effects on (net) methanogenesis, but also for regional‐scale landscape dynamics and indirect temperature effects on carbon cycling.

We anticipate that the projected changes in bottom water temperature will have far‐reaching implications for lake ecosystems this century. Previous studies suggest that bottom water warming may result in, among other things, an alteration to the suitable thermal habitat for many aquatic species (Kraemer et al., 2021), a decrease in dissolved oxygen concentrations at depth due to reduced gas solubility (Jane et al., 2021) and, as a result, nutrient leakage from lake sediments (North et al., 2014), which will have cascading effects on ecosystem productivity. The unknown magnitude of these changes combined with the possibility of aquatic methane emissions becoming a strong climate feedback (Dean et al., 2018) calls for increased efforts to understand carbon cycling in lake and reservoir ecosystems and their sensitivity to environmental change. As our results emphasize, this knowledge gap is particularly pressing for tropical systems where flux measurements remain sparse (Barbosa et al., 2021; Crill et al., 1988; Melack et al., 2004).

## 5 CONCLUSION

Lakes are significant emitters of the potent greenhouse gas methane (CH_4_), and thus are important components of the global CH_4_ budget. CH_4_ is mostly produced in lake sediments, the rate of which is highly temperature dependent. In this work, we show that climate change during the 21st century will result in lakes worldwide experiencing considerable warming of their bottom waters. Low‐latitude lakes will experience the most significant temperature increases, in contrast to the projected latitudinal variation in global air temperatures, because of relatively minor changes in their mixing regime. In turn, our results suggest that CH_4_ production rates will increase considerably (by up to 40%) within a warming world, and that the main part of this increase is accounted for by methanogenesis in low‐latitude lakes.

## CONFLICT OF INTEREST

The authors do not have any competing financial or non‐financial interests to declare.

## CODE AVAILABILITY

The Simstrat‐UoG model is available from the authors, and the latest version of SimStrat can be obtained at https://github.com/Eawag‐AppliedSystemAnalysis/Simstrat/. The GOTM model is available at https://github.com/gotm‐model/code/tree/lake. The MATLAB code used to produce the figures in this paper is available from the corresponding author upon request.

## Supporting information


Appendix S1

## Data Availability

All ISIMIP data and climate model simulations are available at https://data.isimip.org/, and model output specific to the Lake Sector at https://doi.org/10.48364/ISIMIP.931371 for ISIMIP2b lake, global simulations, and https://doi.org/10.48364/ISIMIP.563533 for ISIMIP2b lake, local simulations.
