# Supplementary material for: Global increase in methane production under future warming of lake bottom waters
Source: Glob Chang Biol. 2022 Jun 24;28(18):5427–40. doi: 10.1111/gcb.16298 (PMC9546102; doi:10.1111/gcb.16298)
Supplement: Supplementary file 1 — Appendix S1 [file GCB-28-5427-s001.docx]

**Supplementary Information for**

Global increase in methane production under

future warming of lake bottom waters

Joachim Jansen^*^, R. Iestyn Woolway, Benjamin M. Kraemer, Clément Albergel, David Bastviken, Gesa A. Weyhenmeyer, Rafael Marcé, Sapna Sharma, Sebastian Sobek, Lars Tranvik, Marjorie Perroud, Malgorzata Golub, Tadhg N. Moore, Love Råman Vinnå, Sofia La Fuente, Luke Grant, Don C. Pierson, Wim Thiery, Eleanor Jennings

*To whom correspondence may be addressed.

**Email** [joachim.jansen@ebc.uu.se](mailto:joachim.jansen@ebc.uu.se)

**This file includes:**

Text S1

Figures S1 to S7

Tables S1 to S3

Supplementary References

**Text S1**

*Lake bottom temperature simulations and their limitations*

Our lake temperature simulations, which are available from the ISIMIP Lake Sector, enabled us to capture global scale variability in climate and lake area and depth while accounting for structural lake and climate model uncertainty. When modelling thousands of lakes from around the world, with an ensemble of lake and climate models, certain simplifications inevitably need to be made. In ISIMIP, lake temperatures are simulated with a one-dimensional vertical modelling approach with a simple bathymetry assumption. While the validation results, as well as previous studies, confirm that the lake models can simulate accurately the thermal environment of lakes, notably their bottom temperatures, there are some limitations to consider. Most notably, one-dimensional lake models do not simulate horizontal thermal features in lakes, and thus do not explicitly project the within-lake variations in temperature (Woolway and Merchant 2018). As bottom lake temperatures are typically higher in the shallow near-shore areas compared to the deeper regions, 1D models could therefore potentially overestimate bottom temperatures in the profundal zone but underestimate it in the littoral zone. However, as we apply the same modelling framework globally, this limitation does not challenge the main conclusion of our model study i.e., that the bottom waters of low-latitude lakes will, on average, warm most rapidly this century.

The cylindrical shape of our modelled lakes assumes that, in stratified lakes, the entire sediment area is below the thermocline. In reality, some sediment (e.g., in shallow and flat near-shore areas) will be above the thermocline and thus likely experience higher temperatures, with surface waters following closely the variations in air temperature, and likewise potentially experience greater sediment CH_4_ production rates. Thus, stratifying lakes may experience greater bottom warming, on average, than estimated in this study, with implications for the simulated future CH_4_ production rates. However, we believe that this will not have a considerable influence on our key findings. To demonstrate that the latitudinal variation in future CH_4_ production rates is not influenced by the simple bathymetry assumed in our global simulations, we repeat our future simulations with lake temperature projections for 57 lakes where detailed bathymetry data is available (Supplementary Fig. 7; Supplementary Table 2). That is, a similar modelling approach was used to simulate the 57 lakes but instead of assuming a cylindrical lake, detailed bathymetry was used to describe the lake morphometry within the models. Whilst not directly comparable to the global scale simulations, as many of these lakes are deeper (and thus have cooler bottom temperatures), they clearly demonstrate a latitudinal variation in simulated CH_4_ production rates by the end of the 21^st^ century (Supplementary Fig. 7; Supplementary Table 2). Thus, the simple bathymetry assumption considered in this study is very unlikely to influence the global implications of our findings. Finally, we note that while one-dimensional lake models represent a relatively simplified view of lake responses to climate change, they are used extensively in future climate change impact studies (Shatwell et al. 2019; Woolway and Merchant 2019; Mesman et al. 2020; Maberly et al. 2020; Woolway et al. 2021a; b), and are currently the best tool for projecting global lake thermal responses to future changes in the climate.

Our lake temperature projections are based on representative lakes at a 0.5° latitude-longitude resolution (see Methods). Specifically, the average area and area-weighted mean depth of all lakes within a given 0.5° grid are used to simulate the average lake thermal environment in that location. The dataset used to describe the size distribution of all lakes within each 0.5° grid is based on a 30 arc seconds (~1 km at the equator) resolution product (Kourzeneva 2010). Given the 1 km size restriction of the global product used, our analysis will inevitably be biased to lakes larger than this size threshold. Thus, lakes/ponds smaller than 1 km^2^ are not represented in our global projections. By not accounting for the smallest (and typically shallow) water bodies, which are common at higher latitudes, one might expect the projected change in bottom temperature and thus CH_4_ production rates to be underestimated i.e., as small, shallow lakes could be expected to experience greater change in bottom temperature this century. However, for the shallow high-latitude lakes considered in this study, our projections indicate that many will transition to a more stable stratification regime this century (Fig. 4) and, in turn, experience a somewhat muted response of lake bottom temperature to climatic warming. Indeed, a global synthesis study of summer deep-water temperature changes in 102 lakes (measurements, 1970–2009) suggests that lakes larger than 1 km^2^ have warmed the most, while lakes smaller than that threshold appear to be cooling in summer (Pilla et al. 2020). Furthermore, due to the low historical bottom water temperatures of high-latitude lakes, we expect that relatively small temperature biases due to an overestimation of the average lake size will likely have a minimal effect on the methanogenesis rates calculated (i.e., compared to low-latitude lakes). Thus, the omission of very small lakes is unlikely to influence one of our key findings of greater CH_4_ production in low-latitude lakes.

**Figures S1 to S7**


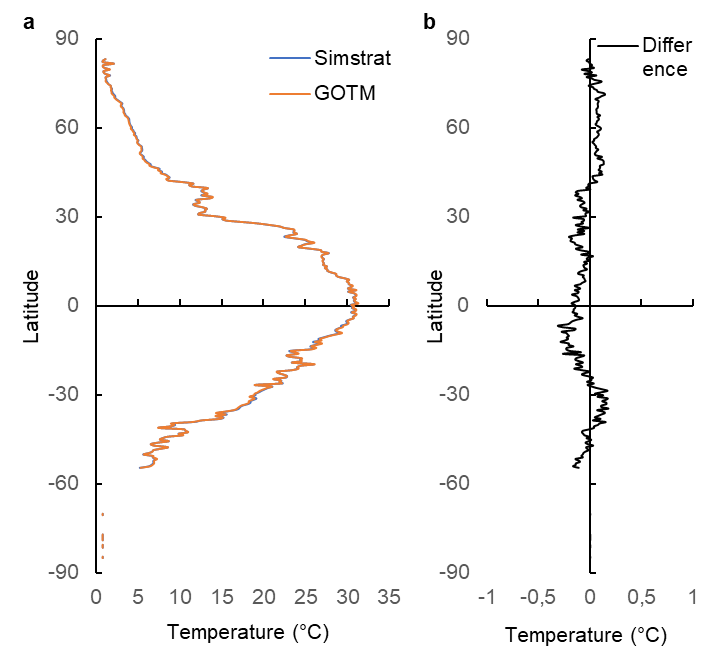


**Supplementary Figure 1 | Comparison of water temperature models GOTM and Simstrat.** Shown is the latitudinal variation of the historical mean lake bottom temperature estimated separately by the 1D models GOTM and Simstrat. Panel a shows a direct comparison between longitudinal averages of the gridded model output. Panel b shows the difference between the models (Simstrat−GOTM). Overall, there is no clear bias and differences are <2% of the ensemble mean.

**
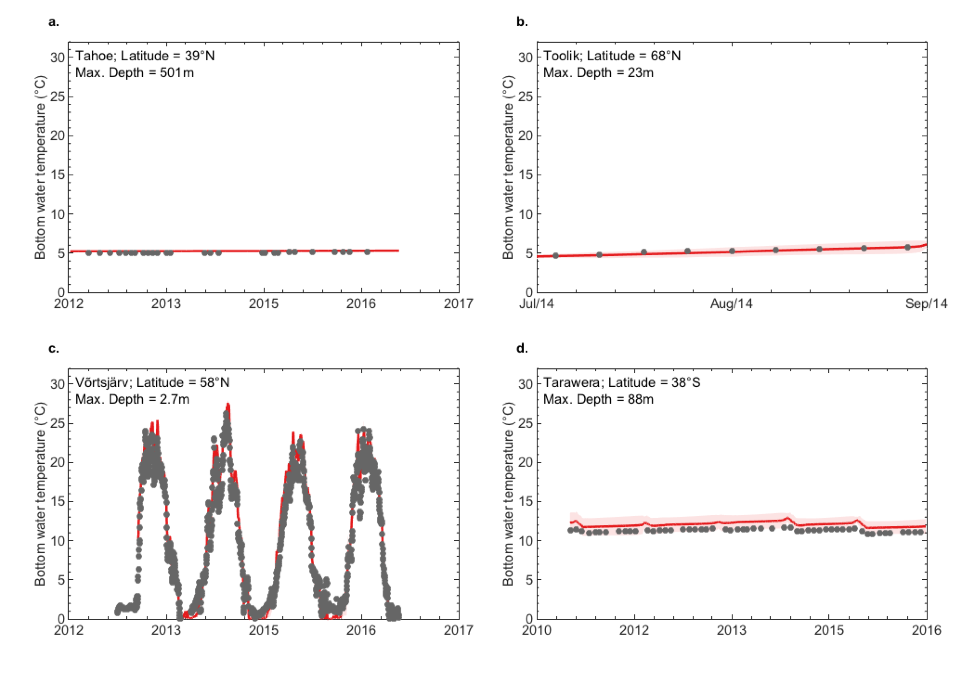
**

**Supplementary Figure 2 | Validation of water temperature simulations in contrasting lakes with available data.** Shown are comparisons between the simulated (red lines) and observed (grey points) bottom water temperatures in four lakes situated across latitudes and morphometric gradients: **(a)** Lake Tahoe (California/Nevada, USA), **(b)** Toolik Lake (Alaska, USA), **(c)** Vōrtsjärv (Estonia), and **(d)** Tarawera (New Zealand). The thick solid red lines show the mean bottom temperature across the lake model ensemble, and the shaded regions represent the range.

Supplementary Figure 3 | Global distribution in the number of mixed days per year across lakes. Shown are (a) the average number of mixed days each year, as well as (b) the latitudinal average. All results represent the mean of the lake-climate model ensemble, and are shown for the 1970–1999 period. A mixed day is defined as when a vertical density gradient of 0.1 kg m^−3^ exists between surface and bottom waters, notably when surface water is less dense than that at depth.


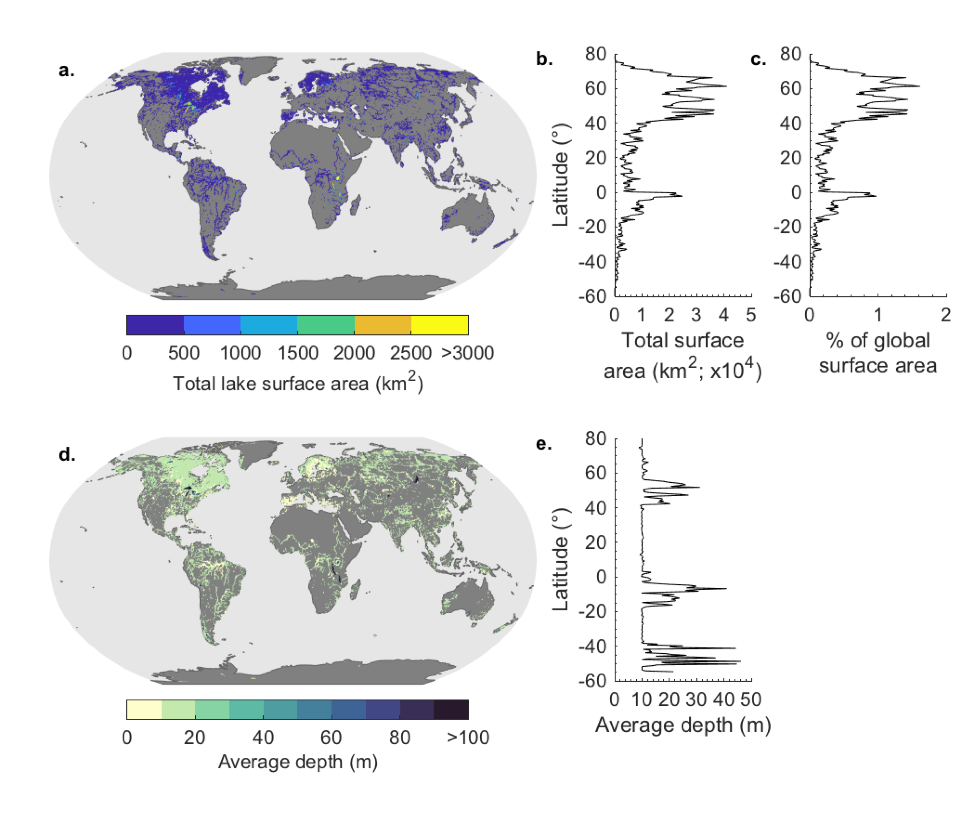


**Supplementary Figure 4 | Global distribution of lake surface area and average depth.** Shown are **(a)** the spatial patterns in total lake surface area per 0.5° grid cell, **(b)** the total surface area as a function of latitude, and **(c)** the percentage of surface area per 0.5° latitude relative to the total global lake surface area. Also shown are (**d)** the spatial patterns in average lake depth per 0.5° grid cell, and **(e)** the average lake depth as a function of latitude. The locations and grid-scale fractions of lakes within each 0.5° grid cell are those used for driving the lake models in ISIMIP2b.


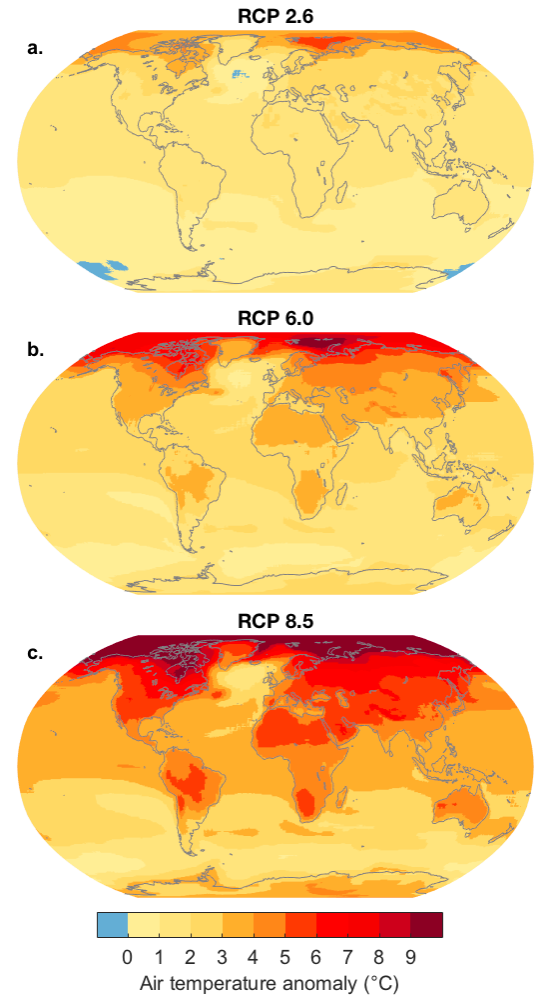


Supplementary Figure 5 | Future projections of global surface air temperature. Shown are the changes in annual average surface air temperature by the end of the 21^st^ century (all years from 2070 to 2099), relative to the 1970-1999 base period average, under (a) Representative Concentration Pathway, RCP, 2.6; (b) RCP 6.0; and (c) RCP 8.5. Air temperature projections represent the computed ensemble mean of the four considered global climate models.

**Supplementary Figure 6 | Future projections of methane production rates based on a random global distribution of *E*_a_'. (a)** The spatial patterns in methane (CH_4_) production rates in lake sediments by the end of the 21^st^ century (2070–2099) under Representative Concentration Pathway (RCP) 8.5 (high-emission), relative to the historical (1970–1999) global average; **(b)** the percent increase in future CH_4_ production rates as a function of latitude under RCP 2.6 (low-emission scenario), 6.0 (medium-high-emission), and 8.5; and **(c)** the absolute change in lake sediment CH_4_ production rates by the end of the 21^st^ century. All results are based on the lake-climate model ensemble. In panels **b** and **c**, the averages are smoothed with a running mean across 2° latitudes. CH_4_ production rates are projected from an Arrhenius-type temperature function with a random global distribution of *E*_a_' among lakes i.e., with site-specific *E*_a_'-values chosen randomly from the distribution of observed values (0.77–1.24 eV).


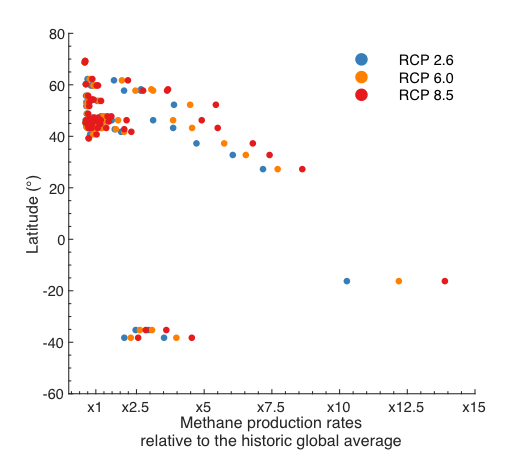


**Supplementary Figure 7 | Future projections of methane production rates in 57 globally distributed lakes.** Shown are the latitudinal variations in the absolute change in CH_4_ production rates in lake sediments by the end of the 21^st^ century (2070–2099). Projections are shown for 57 lakes for which detailed bathymetry information is available. All results are based on the lake-climate model ensemble. See Supplementary Table 2 for a summary of these results.

**Tables S1, S2 and S3**

**Supplementary Table 1 |** **Overview of lake sediment incubation studies used to determine E_a_'**. Analysis after Yvon-Durocher et al. 2014: experimental.unit identifies each unique site, Temp. and rate represent the measured temperature (in temp.units) and methanogenesis rates (in rate.units), respectively, as reported for each incubation experiment. Rates were standardized by subtracting from each rate the experiment-specific intercept at a central temperature value *J*(*T*_C_), where *T*_C_ is defined as the mean of the experiments. *J*(*T*_C_) was computed for each experimental unit via ordinary least squares linear regression of the natural logarithm of the rate (ln.rate) against the inverse of the temperature in K. The standard temperature (std.temp) was computed as $\frac{1}{k_{B}T_{c}} - \frac{1}{k_{B}T}$, where *T* is the temperature in Kelvin and *k*_B_ is the Boltzmann constant (8.62 × 10^−5^ eV K^−1^). The standard rate (std.rate) was computed as $ln\left( J\left( T \right) \right)-ln\left( J\left( T_{C} \right) \right)$.

| **experimental.unit** | **latitude** | **longitude** | **temp** | **rate** | **rate.units** | **ln.rate** | **J(Tc)** | **std.temp** | **std.rate** | **reference** |
| --- | --- | --- | --- | --- | --- | --- | --- | --- | --- | --- |
| Lake Constance | 47°35′N | 9°28′E | 2.03 | 0.17 | nmol CH4 mL^-1 h^-1 | -1.80 | 1.04 | -2.35 | -1.83 | Schulz et al. 1997 |
| Lake Constance | 47°35′N | 9°28′E | 3.71 | 0.21 | nmol CH4 mL^-1 h^-1 | -1.54 | 1.04 | -2.10 | -1.57 | Schulz et al. 1997 |
| Lake Constance | 47°35′N | 9°28′E | 5.60 | 0.22 | nmol CH4 mL^-1 h^-1 | -1.52 | 1.04 | -1.81 | -1.56 | Schulz et al. 1997 |
| Lake Constance | 47°35′N | 9°28′E | 7.03 | 0.22 | nmol CH4 mL^-1 h^-1 | -1.51 | 1.04 | -1.60 | -1.54 | Schulz et al. 1997 |
| Lake Constance | 47°35′N | 9°28′E | 8.85 | 0.20 | nmol CH4 mL^-1 h^-1 | -1.60 | 1.04 | -1.33 | -1.64 | Schulz et al. 1997 |
| Lake Constance | 47°35′N | 9°28′E | 10.13 | 0.25 | nmol CH4 mL^-1 h^-1 | -1.39 | 1.04 | -1.15 | -1.42 | Schulz et al. 1997 |
| Lake Constance | 47°35′N | 9°28′E | 12.03 | 0.25 | nmol CH4 mL^-1 h^-1 | -1.37 | 1.04 | -0.88 | -1.41 | Schulz et al. 1997 |
| Lake Constance | 47°35′N | 9°28′E | 13.45 | 0.30 | nmol CH4 mL^-1 h^-1 | -1.20 | 1.04 | -0.67 | -1.23 | Schulz et al. 1997 |
| Lake Constance | 47°35′N | 9°28′E | 14.95 | 0.37 | nmol CH4 mL^-1 h^-1 | -0.98 | 1.04 | -0.46 | -1.02 | Schulz et al. 1997 |
| Lake Constance | 47°35′N | 9°28′E | 16.52 | 0.45 | nmol CH4 mL^-1 h^-1 | -0.81 | 1.04 | -0.24 | -0.84 | Schulz et al. 1997 |
| Lake Constance | 47°35′N | 9°28′E | 18.09 | 0.50 | nmol CH4 mL^-1 h^-1 | -0.70 | 1.04 | -0.03 | -0.74 | Schulz et al. 1997 |
| Lake Constance | 47°35′N | 9°28′E | 19.48 | 0.80 | nmol CH4 mL^-1 h^-1 | -0.22 | 1.04 | 0.16 | -0.26 | Schulz et al. 1997 |
| Lake Constance | 47°35′N | 9°28′E | 20.95 | 0.80 | nmol CH4 mL^-1 h^-1 | -0.22 | 1.04 | 0.36 | -0.26 | Schulz et al. 1997 |
| Lake Constance | 47°35′N | 9°28′E | 22.65 | 2.22 | nmol CH4 mL^-1 h^-1 | 0.80 | 1.04 | 0.59 | 0.76 | Schulz et al. 1997 |
| Lake Constance | 47°35′N | 9°28′E | 24.19 | 2.59 | nmol CH4 mL^-1 h^-1 | 0.95 | 1.04 | 0.79 | 0.92 | Schulz et al. 1997 |
| Lake Constance | 47°35′N | 9°28′E | 25.97 | 2.41 | nmol CH4 mL^-1 h^-1 | 0.88 | 1.04 | 1.02 | 0.84 | Schulz et al. 1997 |
| Lake Constance | 47°35′N | 9°28′E | 27.46 | 4.40 | nmol CH4 mL^-1 h^-1 | 1.48 | 1.04 | 1.21 | 1.45 | Schulz et al. 1997 |
| Lake Constance | 47°35′N | 9°28′E | 28.98 | 8.59 | nmol CH4 mL^-1 h^-1 | 2.15 | 1.04 | 1.41 | 2.11 | Schulz et al. 1997 |
| Lake Constance | 47°35′N | 9°28′E | 30.82 | 11.94 | nmol CH4 mL^-1 h^-1 | 2.48 | 1.04 | 1.64 | 2.44 | Schulz et al. 1997 |
| Lake Constance | 47°35′N | 9°28′E | 32.30 | 16.76 | nmol CH4 mL^-1 h^-1 | 2.82 | 1.04 | 1.82 | 2.78 | Schulz et al. 1997 |
| Lake Kevätön | 63°6′N | 27°37′E | 5.97 | 8.76 | mg CH4 cm^-2 d^-1 | 2.17 | 29.95 | -1.76 | -1.23 | Liikanen et al. 2002 |
| Lake Kevätön | 63°6′N | 27°37′E | 10.89 | 18.40 | mg CH4 cm^-2 d^-1 | 2.91 | 29.95 | -1.04 | -0.49 | Liikanen et al. 2002 |
| Lake Kevätön | 63°6′N | 27°37′E | 15.54 | 29.24 | mg CH4 cm^-2 d^-1 | 3.38 | 29.95 | -0.38 | -0.02 | Liikanen et al. 2002 |
| Lake Kevätön | 63°6′N | 27°37′E | 22.57 | 35.93 | mg CH4 cm^-2 d^-1 | 3.58 | 29.95 | 0.57 | 0.18 | Liikanen et al. 2002 |
| Lake Mendota - Jan -10m | 43°6′N | 89°25′W | 21.91 | 2.12 | nmol CH4 g^-1 h^-1 | 0.75 | 0.96 | 0.49 | 0.79 | Zeikus and Winfrey 1976 |
| Lake Mendota - Jan -10m | 43°6′N | 89°25′W | 29.76 | 8.24 | nmol CH4 g^-1 h^-1 | 2.11 | 0.96 | 1.51 | 2.15 | Zeikus and Winfrey 1976 |
| Lake Mendota - Jan -10m | 43°6′N | 89°25′W | 36.14 | 33.00 | nmol CH4 g^-1 h^-1 | 3.50 | 0.96 | 2.30 | 3.54 | Zeikus and Winfrey 1976 |
| Lake Mendota - Jan -18m | 43°6′N | 89°25′W | 22.12 | 2.84 | nmol CH4 g^-1 h^-1 | 1.04 | 1.24 | 0.51 | 0.83 | Zeikus and Winfrey 1976 |
| Lake Mendota - Jan -18m | 43°6′N | 89°25′W | 29.76 | 15.99 | nmol CH4 g^-1 h^-1 | 2.77 | 1.24 | 1.51 | 2.56 | Zeikus and Winfrey 1976 |
| Lake Mendota - Jan -18m | 43°6′N | 89°25′W | 34.10 | 32.26 | nmol CH4 g^-1 h^-1 | 3.47 | 1.24 | 2.05 | 3.26 | Zeikus and Winfrey 1976 |
| Lake Mendota - Jan -18m | 43°6′N | 89°25′W | 35.41 | 50.22 | nmol CH4 g^-1 h^-1 | 3.92 | 1.24 | 2.21 | 3.70 | Zeikus and Winfrey 1976 |
| Lake Mendota - Jan -5m | 43°6′N | 89°25′W | 21.95 | 0.82 | nmol CH4 g^-1 h^-1 | -0.19 | 0.56 | 0.49 | 0.39 | Zeikus and Winfrey 1976 |
| Lake Mendota - Jan -5m | 43°6′N | 89°25′W | 29.73 | 1.49 | nmol CH4 g^-1 h^-1 | 0.40 | 0.56 | 1.50 | 0.98 | Zeikus and Winfrey 1976 |
| Lake Mendota - Jan -5m | 43°6′N | 89°25′W | 36.79 | 3.15 | nmol CH4 g^-1 h^-1 | 1.15 | 0.56 | 2.37 | 1.73 | Zeikus and Winfrey 1976 |
| Lake Mendota - May -10m | 43°6′N | 89°25′W | 3.35 | 0.86 | nmol CH4 g^-1 h^-1 | -0.15 | 6.18 | -2.15 | -1.97 | Zeikus and Winfrey 1976 |
| Lake Mendota - May -10m | 43°6′N | 89°25′W | 19.27 | 10.63 | nmol CH4 g^-1 h^-1 | 2.36 | 6.18 | 0.13 | 0.54 | Zeikus and Winfrey 1976 |
| Lake Mendota - May -10m | 43°6′N | 89°25′W | 29.20 | 19.52 | nmol CH4 g^-1 h^-1 | 2.97 | 6.18 | 1.43 | 1.15 | Zeikus and Winfrey 1976 |
| Lake Mendota - May -10m | 43°6′N | 89°25′W | 35.72 | 31.55 | nmol CH4 g^-1 h^-1 | 3.45 | 6.18 | 2.24 | 1.63 | Zeikus and Winfrey 1976 |
| Lake Mendota - May -15m | 43°6′N | 89°25′W | 3.53 | 4.02 | nmol CH4 g^-1 h^-1 | 1.39 | 11.93 | -2.13 | -1.09 | Zeikus and Winfrey 1976 |
| Lake Mendota - May -15m | 43°6′N | 89°25′W | 19.22 | 13.66 | nmol CH4 g^-1 h^-1 | 2.61 | 11.93 | 0.12 | 0.14 | Zeikus and Winfrey 1976 |
| Lake Mendota - May -15m | 43°6′N | 89°25′W | 28.88 | 24.37 | nmol CH4 g^-1 h^-1 | 3.19 | 11.93 | 1.39 | 0.71 | Zeikus and Winfrey 1976 |
| Lake Mendota - May -15m | 43°6′N | 89°25′W | 35.45 | 33.37 | nmol CH4 g^-1 h^-1 | 3.51 | 11.93 | 2.21 | 1.03 | Zeikus and Winfrey 1976 |
| Lake Mendota - May -5m | 43°6′N | 89°25′W | 3.60 | 0.02 | nmol CH4 g^-1 h^-1 | -4.14 | 0.78 | -2.11 | -3.89 | Zeikus and Winfrey 1976 |
| Lake Mendota - May -5m | 43°6′N | 89°25′W | 18.45 | 2.38 | nmol CH4 g^-1 h^-1 | 0.87 | 0.78 | 0.02 | 1.12 | Zeikus and Winfrey 1976 |
| Lake Mendota - May -5m | 43°6′N | 89°25′W | 29.41 | 7.77 | nmol CH4 g^-1 h^-1 | 2.05 | 0.78 | 1.46 | 2.30 | Zeikus and Winfrey 1976 |
| Lake Mendota - May -5m | 43°6′N | 89°25′W | 36.43 | 18.10 | nmol CH4 g^-1 h^-1 | 2.90 | 0.78 | 2.33 | 3.15 | Zeikus and Winfrey 1976 |
| Funbosjön | 59°51′39″N | 17°51′81″E | 4.00 | 0.14 | µmol CH4 g_dw^-1 day^-1 | -2.00 | 0.56 | -2.05 | -1.42 | Duc et al. 2010 |
| Funbosjön | 59°51′39″N | 17°51′81″E | 4.00 | 0.03 | µmol CH4 g_dw^-1 day^-1 | -3.47 | 0.56 | -2.05 | -2.89 | Duc et al. 2010 |
| Funbosjön | 59°51′39″N | 17°51′81″E | 4.00 | 0.09 | µmol CH4 g_dw^-1 day^-1 | -2.44 | 0.56 | -2.05 | -1.86 | Duc et al. 2010 |
| Funbosjön | 59°51′39″N | 17°51′81″E | 10.00 | 0.18 | µmol CH4 g_dw^-1 day^-1 | -1.70 | 0.56 | -1.17 | -1.13 | Duc et al. 2010 |
| Funbosjön | 59°51′39″N | 17°51′81″E | 10.00 | 0.20 | µmol CH4 g_dw^-1 day^-1 | -1.60 | 0.56 | -1.17 | -1.03 | Duc et al. 2010 |
| Funbosjön | 59°51′39″N | 17°51′81″E | 10.00 | 0.17 | µmol CH4 g_dw^-1 day^-1 | -1.75 | 0.56 | -1.17 | -1.17 | Duc et al. 2010 |
| Funbosjön | 59°51′39″N | 17°51′81″E | 20.00 | 0.98 | µmol CH4 g_dw^-1 day^-1 | -0.02 | 0.56 | 0.23 | 0.56 | Duc et al. 2010 |
| Funbosjön | 59°51′39″N | 17°51′81″E | 20.00 | 0.82 | µmol CH4 g_dw^-1 day^-1 | -0.20 | 0.56 | 0.23 | 0.38 | Duc et al. 2010 |
| Funbosjön | 59°51′39″N | 17°51′81″E | 20.00 | 0.96 | µmol CH4 g_dw^-1 day^-1 | -0.04 | 0.56 | 0.23 | 0.53 | Duc et al. 2010 |
| Funbosjön | 59°51′39″N | 17°51′81″E | 30.00 | 2.09 | µmol CH4 g_dw^-1 day^-1 | 0.74 | 0.56 | 1.54 | 1.32 | Duc et al. 2010 |
| Funbosjön | 59°51′39″N | 17°51′81″E | 30.00 | 1.96 | µmol CH4 g_dw^-1 day^-1 | 0.67 | 0.56 | 1.54 | 1.25 | Duc et al. 2010 |
| Funbosjön | 59°51′39″N | 17°51′81″E | 30.00 | 2.06 | µmol CH4 g_dw^-1 day^-1 | 0.72 | 0.56 | 1.54 | 1.30 | Duc et al. 2010 |
| Lilla Sången | 59°54′10″N | 15°23′37″E | 4.00 | 0.00 | µmol CH4 g_dw^-1 day^-1 | -6.28 | 0.01 | -2.05 | -1.52 | Duc et al. 2010 |
| Lilla Sången | 59°54′10″N | 15°23′37″E | 4.00 | 0.00 | µmol CH4 g_dw^-1 day^-1 | -6.80 | 0.01 | -2.05 | -2.04 | Duc et al. 2010 |
| Lilla Sången | 59°54′10″N | 15°23′37″E | 4.00 | 0.00 | µmol CH4 g_dw^-1 day^-1 | -5.79 | 0.01 | -2.05 | -1.03 | Duc et al. 2010 |
| Lilla Sången | 59°54′10″N | 15°23′37″E | 10.00 | 0.00 | µmol CH4 g_dw^-1 day^-1 | -7.05 | 0.01 | -1.17 | -2.30 | Duc et al. 2010 |
| Lilla Sången | 59°54′10″N | 15°23′37″E | 10.00 | 0.00 | µmol CH4 g_dw^-1 day^-1 | -5.41 | 0.01 | -1.17 | -0.65 | Duc et al. 2010 |
| Lilla Sången | 59°54′10″N | 15°23′37″E | 10.00 | 0.00 | µmol CH4 g_dw^-1 day^-1 | -6.25 | 0.01 | -1.17 | -1.50 | Duc et al. 2010 |
| Lilla Sången | 59°54′10″N | 15°23′37″E | 20.00 | 0.02 | µmol CH4 g_dw^-1 day^-1 | -4.14 | 0.01 | 0.23 | 0.62 | Duc et al. 2010 |
| Lilla Sången | 59°54′10″N | 15°23′37″E | 20.00 | 0.01 | µmol CH4 g_dw^-1 day^-1 | -4.93 | 0.01 | 0.23 | -0.18 | Duc et al. 2010 |
| Lilla Sången | 59°54′10″N | 15°23′37″E | 20.00 | 0.01 | µmol CH4 g_dw^-1 day^-1 | -4.40 | 0.01 | 0.23 | 0.36 | Duc et al. 2010 |
| Lilla Sången | 59°54′10″N | 15°23′37″E | 30.00 | 0.04 | µmol CH4 g_dw^-1 day^-1 | -3.16 | 0.01 | 1.54 | 1.59 | Duc et al. 2010 |
| Lilla Sången | 59°54′10″N | 15°23′37″E | 30.00 | 0.03 | µmol CH4 g_dw^-1 day^-1 | -3.48 | 0.01 | 1.54 | 1.27 | Duc et al. 2010 |
| Lilla Sången | 59°54′10″N | 15°23′37″E | 30.00 | 0.04 | µmol CH4 g_dw^-1 day^-1 | -3.28 | 0.01 | 1.54 | 1.48 | Duc et al. 2010 |
| Limmaren | 59°43′43″N | 18°45′2″E | 4.00 | 0.04 | µmol CH4 g_dw^-1 day^-1 | -3.16 | 0.15 | -2.05 | -1.27 | Duc et al. 2010 |
| Limmaren | 59°43′43″N | 18°45′2″E | 4.00 | 0.04 | µmol CH4 g_dw^-1 day^-1 | -3.22 | 0.15 | -2.05 | -1.33 | Duc et al. 2010 |
| Limmaren | 59°43′43″N | 18°45′2″E | 4.00 | 0.04 | µmol CH4 g_dw^-1 day^-1 | -3.23 | 0.15 | -2.05 | -1.35 | Duc et al. 2010 |
| Limmaren | 59°43′43″N | 18°45′2″E | 10.00 | 0.06 | µmol CH4 g_dw^-1 day^-1 | -2.75 | 0.15 | -1.17 | -0.87 | Duc et al. 2010 |
| Limmaren | 59°43′43″N | 18°45′2″E | 10.00 | 0.06 | µmol CH4 g_dw^-1 day^-1 | -2.75 | 0.15 | -1.17 | -0.87 | Duc et al. 2010 |
| Limmaren | 59°43′43″N | 18°45′2″E | 10.00 | 0.07 | µmol CH4 g_dw^-1 day^-1 | -2.61 | 0.15 | -1.17 | -0.72 | Duc et al. 2010 |
| Limmaren | 59°43′43″N | 18°45′2″E | 20.00 | 0.21 | µmol CH4 g_dw^-1 day^-1 | -1.55 | 0.15 | 0.23 | 0.33 | Duc et al. 2010 |
| Limmaren | 59°43′43″N | 18°45′2″E | 20.00 | 0.19 | µmol CH4 g_dw^-1 day^-1 | -1.67 | 0.15 | 0.23 | 0.21 | Duc et al. 2010 |
| Limmaren | 59°43′43″N | 18°45′2″E | 20.00 | 0.20 | µmol CH4 g_dw^-1 day^-1 | -1.63 | 0.15 | 0.23 | 0.25 | Duc et al. 2010 |
| Limmaren | 59°43′43″N | 18°45′2″E | 30.00 | 0.37 | µmol CH4 g_dw^-1 day^-1 | -1.00 | 0.15 | 1.54 | 0.89 | Duc et al. 2010 |
| Limmaren | 59°43′43″N | 18°45′2″E | 30.00 | 0.38 | µmol CH4 g_dw^-1 day^-1 | -0.96 | 0.15 | 1.54 | 0.93 | Duc et al. 2010 |
| Limmaren | 59°43′43″N | 18°45′2″E | 30.00 | 0.41 | µmol CH4 g_dw^-1 day^-1 | -0.89 | 0.15 | 1.54 | 0.99 | Duc et al. 2010 |
| Ljustjärn | 59°55′21″N | 15°27′13″E | 4.00 | 0.01 | µmol CH4 g_dw^-1 day^-1 | -4.96 | 0.04 | -2.05 | -1.61 | Duc et al. 2010 |
| Ljustjärn | 59°55′21″N | 15°27′13″E | 4.00 | 0.01 | µmol CH4 g_dw^-1 day^-1 | -4.62 | 0.04 | -2.05 | -1.27 | Duc et al. 2010 |
| Ljustjärn | 59°55′21″N | 15°27′13″E | 4.00 | 0.00 | µmol CH4 g_dw^-1 day^-1 | -5.95 | 0.04 | -2.05 | -2.60 | Duc et al. 2010 |
| Ljustjärn | 59°55′21″N | 15°27′13″E | 10.00 | 0.00 | µmol CH4 g_dw^-1 day^-1 | -5.37 | 0.04 | -1.17 | -2.02 | Duc et al. 2010 |
| Ljustjärn | 59°55′21″N | 15°27′13″E | 10.00 | 0.01 | µmol CH4 g_dw^-1 day^-1 | -4.84 | 0.04 | -1.17 | -1.49 | Duc et al. 2010 |
| Ljustjärn | 59°55′21″N | 15°27′13″E | 10.00 | 0.01 | µmol CH4 g_dw^-1 day^-1 | -5.06 | 0.04 | -1.17 | -1.71 | Duc et al. 2010 |
| Ljustjärn | 59°55′21″N | 15°27′13″E | 20.00 | 0.04 | µmol CH4 g_dw^-1 day^-1 | -3.27 | 0.04 | 0.23 | 0.09 | Duc et al. 2010 |
| Ljustjärn | 59°55′21″N | 15°27′13″E | 20.00 | 0.02 | µmol CH4 g_dw^-1 day^-1 | -3.89 | 0.04 | 0.23 | -0.54 | Duc et al. 2010 |
| Ljustjärn | 59°55′21″N | 15°27′13″E | 20.00 | 0.02 | µmol CH4 g_dw^-1 day^-1 | -3.71 | 0.04 | 0.23 | -0.36 | Duc et al. 2010 |
| Ljustjärn | 59°55′21″N | 15°27′13″E | 30.00 | 0.35 | µmol CH4 g_dw^-1 day^-1 | -1.06 | 0.04 | 1.54 | 2.30 | Duc et al. 2010 |
| Ljustjärn | 59°55′21″N | 15°27′13″E | 30.00 | 0.23 | µmol CH4 g_dw^-1 day^-1 | -1.48 | 0.04 | 1.54 | 1.88 | Duc et al. 2010 |
| Ljustjärn | 59°55′21″N | 15°27′13″E | 30.00 | 0.38 | µmol CH4 g_dw^-1 day^-1 | -0.97 | 0.04 | 1.54 | 2.38 | Duc et al. 2010 |
| Lötsjön | 59°52′1″N | 17°56′51″E | 4.00 | 0.07 | µmol CH4 g_dw^-1 day^-1 | -2.68 | 0.77 | -2.05 | -2.42 | Duc et al. 2010 |
| Lötsjön | 59°52′1″N | 17°56′51″E | 4.00 | 0.06 | µmol CH4 g_dw^-1 day^-1 | -2.87 | 0.77 | -2.05 | -2.61 | Duc et al. 2010 |
| Lötsjön | 59°52′1″N | 17°56′51″E | 4.00 | 0.08 | µmol CH4 g_dw^-1 day^-1 | -2.50 | 0.77 | -2.05 | -2.23 | Duc et al. 2010 |
| Lötsjön | 59°52′1″N | 17°56′51″E | 10.00 | 0.19 | µmol CH4 g_dw^-1 day^-1 | -1.64 | 0.77 | -1.17 | -1.38 | Duc et al. 2010 |
| Lötsjön | 59°52′1″N | 17°56′51″E | 10.00 | 0.14 | µmol CH4 g_dw^-1 day^-1 | -1.97 | 0.77 | -1.17 | -1.71 | Duc et al. 2010 |
| Lötsjön | 59°52′1″N | 17°56′51″E | 10.00 | 0.18 | µmol CH4 g_dw^-1 day^-1 | -1.73 | 0.77 | -1.17 | -1.47 | Duc et al. 2010 |
| Lötsjön | 59°52′1″N | 17°56′51″E | 20.00 | 1.36 | µmol CH4 g_dw^-1 day^-1 | 0.31 | 0.77 | 0.23 | 0.57 | Duc et al. 2010 |
| Lötsjön | 59°52′1″N | 17°56′51″E | 20.00 | 1.36 | µmol CH4 g_dw^-1 day^-1 | 0.31 | 0.77 | 0.23 | 0.57 | Duc et al. 2010 |
| Lötsjön | 59°52′1″N | 17°56′51″E | 20.00 | 1.38 | µmol CH4 g_dw^-1 day^-1 | 0.32 | 0.77 | 0.23 | 0.59 | Duc et al. 2010 |
| Lötsjön | 59°52′1″N | 17°56′51″E | 30.00 | 3.88 | µmol CH4 g_dw^-1 day^-1 | 1.35 | 0.77 | 1.54 | 1.62 | Duc et al. 2010 |
| Lötsjön | 59°52′1″N | 17°56′51″E | 30.00 | 4.20 | µmol CH4 g_dw^-1 day^-1 | 1.43 | 0.77 | 1.54 | 1.70 | Duc et al. 2010 |
| Lötsjön | 59°52′1″N | 17°56′51″E | 30.00 | 3.90 | µmol CH4 g_dw^-1 day^-1 | 1.36 | 0.77 | 1.54 | 1.62 | Duc et al. 2010 |
| Oppsveten | 60°00′54″N | 15°28′38″E | 4.00 | 0.02 | µmol CH4 g_dw^-1 day^-1 | -3.82 | 0.06 | -2.05 | -1.05 | Duc et al. 2010 |
| Oppsveten | 60°00′54″N | 15°28′38″E | 4.00 | 0.02 | µmol CH4 g_dw^-1 day^-1 | -3.96 | 0.06 | -2.05 | -1.20 | Duc et al. 2010 |
| Oppsveten | 60°00′54″N | 15°28′38″E | 4.00 | 0.02 | µmol CH4 g_dw^-1 day^-1 | -4.17 | 0.06 | -2.05 | -1.41 | Duc et al. 2010 |
| Oppsveten | 60°00′54″N | 15°28′38″E | 10.00 | 0.01 | µmol CH4 g_dw^-1 day^-1 | -4.31 | 0.06 | -1.17 | -1.54 | Duc et al. 2010 |
| Oppsveten | 60°00′54″N | 15°28′38″E | 10.00 | 0.02 | µmol CH4 g_dw^-1 day^-1 | -3.70 | 0.06 | -1.17 | -0.94 | Duc et al. 2010 |
| Oppsveten | 60°00′54″N | 15°28′38″E | 10.00 | 0.02 | µmol CH4 g_dw^-1 day^-1 | -4.19 | 0.06 | -1.17 | -1.43 | Duc et al. 2010 |
| Oppsveten | 60°00′54″N | 15°28′38″E | 20.00 | 0.04 | µmol CH4 g_dw^-1 day^-1 | -3.17 | 0.06 | 0.23 | -0.41 | Duc et al. 2010 |
| Oppsveten | 60°00′54″N | 15°28′38″E | 20.00 | 0.03 | µmol CH4 g_dw^-1 day^-1 | -3.65 | 0.06 | 0.23 | -0.88 | Duc et al. 2010 |
| Oppsveten | 60°00′54″N | 15°28′38″E | 20.00 | 0.02 | µmol CH4 g_dw^-1 day^-1 | -3.74 | 0.06 | 0.23 | -0.98 | Duc et al. 2010 |
| Oppsveten | 60°00′54″N | 15°28′38″E | 30.00 | 0.60 | µmol CH4 g_dw^-1 day^-1 | -0.52 | 0.06 | 1.54 | 2.25 | Duc et al. 2010 |
| Oppsveten | 60°00′54″N | 15°28′38″E | 30.00 | 0.39 | µmol CH4 g_dw^-1 day^-1 | -0.94 | 0.06 | 1.54 | 1.82 | Duc et al. 2010 |
| Oppsveten | 60°00′54″N | 15°28′38″E | 30.00 | 0.46 | µmol CH4 g_dw^-1 day^-1 | -0.78 | 0.06 | 1.54 | 1.98 | Duc et al. 2010 |
| Svarttjärn | 59°53′25″N | 15°15′27″E | 4.00 | 0.01 | µmol CH4 g_dw^-1 day^-1 | -4.82 | 0.07 | -2.05 | -2.15 | Duc et al. 2010 |
| Svarttjärn | 59°53′25″N | 15°15′27″E | 4.00 | 0.01 | µmol CH4 g_dw^-1 day^-1 | -4.39 | 0.07 | -2.05 | -1.72 | Duc et al. 2010 |
| Svarttjärn | 59°53′25″N | 15°15′27″E | 4.00 | 0.01 | µmol CH4 g_dw^-1 day^-1 | -4.67 | 0.07 | -2.05 | -2.00 | Duc et al. 2010 |
| Svarttjärn | 59°53′25″N | 15°15′27″E | 10.00 | 0.01 | µmol CH4 g_dw^-1 day^-1 | -4.49 | 0.07 | -1.17 | -1.82 | Duc et al. 2010 |
| Svarttjärn | 59°53′25″N | 15°15′27″E | 10.00 | 0.01 | µmol CH4 g_dw^-1 day^-1 | -4.68 | 0.07 | -1.17 | -2.02 | Duc et al. 2010 |
| Svarttjärn | 59°53′25″N | 15°15′27″E | 10.00 | 0.01 | µmol CH4 g_dw^-1 day^-1 | -4.89 | 0.07 | -1.17 | -2.22 | Duc et al. 2010 |
| Svarttjärn | 59°53′25″N | 15°15′27″E | 20.00 | 0.06 | µmol CH4 g_dw^-1 day^-1 | -2.88 | 0.07 | 0.23 | -0.21 | Duc et al. 2010 |
| Svarttjärn | 59°53′25″N | 15°15′27″E | 20.00 | 0.03 | µmol CH4 g_dw^-1 day^-1 | -3.68 | 0.07 | 0.23 | -1.01 | Duc et al. 2010 |
| Svarttjärn | 59°53′25″N | 15°15′27″E | 20.00 | 0.04 | µmol CH4 g_dw^-1 day^-1 | -3.18 | 0.07 | 0.23 | -0.51 | Duc et al. 2010 |
| Svarttjärn | 59°53′25″N | 15°15′27″E | 30.00 | 0.97 | µmol CH4 g_dw^-1 day^-1 | -0.03 | 0.07 | 1.54 | 2.63 | Duc et al. 2010 |
| Svarttjärn | 59°53′25″N | 15°15′27″E | 30.00 | 0.94 | µmol CH4 g_dw^-1 day^-1 | -0.06 | 0.07 | 1.54 | 2.61 | Duc et al. 2010 |
| Svarttjärn | 59°53′25″N | 15°15′27″E | 30.00 | 1.10 | µmol CH4 g_dw^-1 day^-1 | 0.10 | 0.07 | 1.54 | 2.77 | Duc et al. 2010 |
| Valloxen | 59°44′30″N | 17°49′41″E | 4.00 | 0.01 | µmol CH4 g_dw^-1 day^-1 | -4.43 | 0.09 | -2.05 | -2.07 | Duc et al. 2010 |
| Valloxen | 59°44′30″N | 17°49′41″E | 4.00 | 0.02 | µmol CH4 g_dw^-1 day^-1 | -4.17 | 0.09 | -2.05 | -1.81 | Duc et al. 2010 |
| Valloxen | 59°44′30″N | 17°49′41″E | 4.00 | 0.01 | µmol CH4 g_dw^-1 day^-1 | -4.77 | 0.09 | -2.05 | -2.41 | Duc et al. 2010 |
| Valloxen | 59°44′30″N | 17°49′41″E | 10.00 | 0.03 | µmol CH4 g_dw^-1 day^-1 | -3.54 | 0.09 | -1.17 | -1.18 | Duc et al. 2010 |
| Valloxen | 59°44′30″N | 17°49′41″E | 10.00 | 0.02 | µmol CH4 g_dw^-1 day^-1 | -3.88 | 0.09 | -1.17 | -1.52 | Duc et al. 2010 |
| Valloxen | 59°44′30″N | 17°49′41″E | 10.00 | 0.02 | µmol CH4 g_dw^-1 day^-1 | -3.83 | 0.09 | -1.17 | -1.46 | Duc et al. 2010 |
| Valloxen | 59°44′30″N | 17°49′41″E | 20.00 | 0.14 | µmol CH4 g_dw^-1 day^-1 | -1.98 | 0.09 | 0.23 | 0.38 | Duc et al. 2010 |
| Valloxen | 59°44′30″N | 17°49′41″E | 20.00 | 0.16 | µmol CH4 g_dw^-1 day^-1 | -1.84 | 0.09 | 0.23 | 0.52 | Duc et al. 2010 |
| Valloxen | 59°44′30″N | 17°49′41″E | 20.00 | 0.15 | µmol CH4 g_dw^-1 day^-1 | -1.86 | 0.09 | 0.23 | 0.50 | Duc et al. 2010 |
| Valloxen | 59°44′30″N | 17°49′41″E | 30.00 | 0.38 | µmol CH4 g_dw^-1 day^-1 | -0.98 | 0.09 | 1.54 | 1.38 | Duc et al. 2010 |
| Valloxen | 59°44′30″N | 17°49′41″E | 30.00 | 0.48 | µmol CH4 g_dw^-1 day^-1 | -0.73 | 0.09 | 1.54 | 1.63 | Duc et al. 2010 |
| Valloxen | 59°44′30″N | 17°49′41″E | 30.00 | 0.42 | µmol CH4 g_dw^-1 day^-1 | -0.87 | 0.09 | 1.54 | 1.49 | Duc et al. 2010 |
| Amazon 1 | 2°26'S | 54°46'W | 4.3 | 1.1 | µg C L^-1 day^-1 | 0.11 | 11.75 | -2.01 | -2.36 | Marotta et al. 2014 |
| Amazon 1 | 2°26'S | 54°46'W | 10.0 | 10.6 | µg C L^-1 day^-1 | 2.36 | 11.75 | -1.17 | -0.11 | Marotta et al. 2014 |
| Amazon 1 | 2°26'S | 54°46'W | 13.7 | 6.2 | µg C L^-1 day^-1 | 1.82 | 11.75 | -0.64 | -0.64 | Marotta et al. 2014 |
| Amazon 1 | 2°26'S | 54°46'W | 19.3 | 6.4 | µg C L^-1 day^-1 | 1.85 | 11.75 | 0.14 | -0.61 | Marotta et al. 2014 |
| Amazon 1 | 2°26'S | 54°46'W | 29.1 | 42.5 | µg C L^-1 day^-1 | 3.75 | 11.75 | 1.42 | 1.29 | Marotta et al. 2014 |
| Amazon 1 | 2°26'S | 54°46'W | 40.5 | 229.8 | µg C L^-1 day^-1 | 5.44 | 11.75 | 2.82 | 2.97 | Marotta et al. 2014 |
| Amazon 2 | 2°31'S | 54°29'W | 4.3 | 2.0 | µg C L^-1 day^-1 | 0.70 | 3.54 | -2.01 | -0.57 | Marotta et al. 2014 |
| Amazon 2 | 2°31'S | 54°29'W | 10.0 | 2.2 | µg C L^-1 day^-1 | 0.78 | 3.54 | -1.17 | -0.48 | Marotta et al. 2014 |
| Amazon 2 | 2°31'S | 54°29'W | 13.7 | 2.4 | µg C L^-1 day^-1 | 0.87 | 3.54 | -0.64 | -0.40 | Marotta et al. 2014 |
| Amazon 2 | 2°31'S | 54°29'W | 19.3 | 2.4 | µg C L^-1 day^-1 | 0.89 | 3.54 | 0.14 | -0.37 | Marotta et al. 2014 |
| Amazon 2 | 2°31'S | 54°29'W | 29.1 | 2.7 | µg C L^-1 day^-1 | 0.98 | 3.54 | 1.42 | -0.29 | Marotta et al. 2014 |
| Amazon 2 | 2°31'S | 54°29'W | 40.5 | 39.1 | µg C L^-1 day^-1 | 3.67 | 3.54 | 2.82 | 2.40 | Marotta et al. 2014 |
| Amazon 3 | 2°31'S | 54°25'W | 10.0 | 4.4 | µg C L^-1 day^-1 | 1.48 | 11.35 | -1.17 | -0.95 | Marotta et al. 2014 |
| Amazon 3 | 2°31'S | 54°25'W | 13.7 | 4.0 | µg C L^-1 day^-1 | 1.39 | 11.35 | -0.64 | -1.04 | Marotta et al. 2014 |
| Amazon 3 | 2°31'S | 54°25'W | 19.3 | 16.2 | µg C L^-1 day^-1 | 2.78 | 11.35 | 0.14 | 0.35 | Marotta et al. 2014 |
| Amazon 3 | 2°31'S | 54°25'W | 29.1 | 54.7 | µg C L^-1 day^-1 | 4.00 | 11.35 | 1.42 | 1.57 | Marotta et al. 2014 |
| Amazon 3 | 2°31'S | 54°25'W | 40.5 | 137.3 | µg C L^-1 day^-1 | 4.92 | 11.35 | 2.82 | 2.49 | Marotta et al. 2014 |
| Amazon 4 | 2°27'S | 54°5'W | 10.0 | 4.6 | µg C L^-1 day^-1 | 1.53 | 7.42 | -1.17 | -0.48 | Marotta et al. 2014 |
| Amazon 4 | 2°27'S | 54°5'W | 13.7 | 6.2 | µg C L^-1 day^-1 | 1.82 | 7.42 | -0.64 | -0.18 | Marotta et al. 2014 |
| Amazon 4 | 2°27'S | 54°5'W | 19.3 | 20.3 | µg C L^-1 day^-1 | 3.01 | 7.42 | 0.14 | 1.00 | Marotta et al. 2014 |
| Amazon 4 | 2°27'S | 54°5'W | 29.1 | 2.7 | µg C L^-1 day^-1 | 0.98 | 7.42 | 1.42 | -1.03 | Marotta et al. 2014 |
| Amazon 4 | 2°27'S | 54°5'W | 40.5 | 30.1 | µg C L^-1 day^-1 | 3.40 | 7.42 | 2.82 | 1.40 | Marotta et al. 2014 |
| Amazon 5 | 2°28'S | 54°6'W | 4.3 | 2.6 | µg C L^-1 day^-1 | 0.97 | 4.88 | -2.01 | -0.62 | Marotta et al. 2014 |
| Amazon 5 | 2°28'S | 54°6'W | 10.0 | 1.8 | µg C L^-1 day^-1 | 0.58 | 4.88 | -1.17 | -1.01 | Marotta et al. 2014 |
| Amazon 5 | 2°28'S | 54°6'W | 13.7 | 0.7 | µg C L^-1 day^-1 | -0.31 | 4.88 | -0.64 | -1.89 | Marotta et al. 2014 |
| Amazon 5 | 2°28'S | 54°6'W | 19.3 | 2.3 | µg C L^-1 day^-1 | 0.85 | 4.88 | 0.14 | -0.74 | Marotta et al. 2014 |
| Amazon 5 | 2°28'S | 54°6'W | 29.1 | 28.9 | µg C L^-1 day^-1 | 3.36 | 4.88 | 1.42 | 1.78 | Marotta et al. 2014 |
| Amazon 5 | 2°28'S | 54°6'W | 40.5 | 97.4 | µg C L^-1 day^-1 | 4.58 | 4.88 | 2.82 | 2.99 | Marotta et al. 2014 |
| Amazon 6 | 2°29'S | 54°5'W | 4.3 | 3.9 | µg C L^-1 day^-1 | 1.35 | 22.45 | -2.01 | -1.76 | Marotta et al. 2014 |
| Amazon 6 | 2°29'S | 54°5'W | 10.0 | 9.7 | µg C L^-1 day^-1 | 2.27 | 22.45 | -1.17 | -0.84 | Marotta et al. 2014 |
| Amazon 6 | 2°29'S | 54°5'W | 13.7 | 2.5 | µg C L^-1 day^-1 | 0.91 | 22.45 | -0.64 | -2.21 | Marotta et al. 2014 |
| Amazon 6 | 2°29'S | 54°5'W | 19.3 | 38.9 | µg C L^-1 day^-1 | 3.66 | 22.45 | 0.14 | 0.55 | Marotta et al. 2014 |
| Amazon 6 | 2°29'S | 54°5'W | 29.1 | 161.1 | µg C L^-1 day^-1 | 5.08 | 22.45 | 1.42 | 1.97 | Marotta et al. 2014 |
| Amazon 6 | 2°29'S | 54°5'W | 40.5 | 403.4 | µg C L^-1 day^-1 | 6.00 | 22.45 | 2.82 | 2.89 | Marotta et al. 2014 |
| Amazon 7 | 2°25'S | 54°16'W | 4.3 | 12.6 | µg C L^-1 day^-1 | 2.53 | 10.98 | -2.01 | 0.14 | Marotta et al. 2014 |
| Amazon 7 | 2°25'S | 54°16'W | 10.0 | 5.3 | µg C L^-1 day^-1 | 1.67 | 10.98 | -1.17 | -0.73 | Marotta et al. 2014 |
| Amazon 7 | 2°25'S | 54°16'W | 13.7 | 0.8 | µg C L^-1 day^-1 | -0.20 | 10.98 | -0.64 | -2.59 | Marotta et al. 2014 |
| Amazon 7 | 2°25'S | 54°16'W | 19.3 | 6.9 | µg C L^-1 day^-1 | 1.93 | 10.98 | 0.14 | -0.47 | Marotta et al. 2014 |
| Amazon 7 | 2°25'S | 54°16'W | 29.1 | 48.4 | µg C L^-1 day^-1 | 3.88 | 10.98 | 1.42 | 1.48 | Marotta et al. 2014 |
| Amazon 7 | 2°25'S | 54°16'W | 40.5 | 143.9 | µg C L^-1 day^-1 | 4.97 | 10.98 | 2.82 | 2.57 | Marotta et al. 2014 |
| Amazon 8 | 2°26'S | 54°54'W | 10.0 | 3.8 | µg C L^-1 day^-1 | 1.34 | 6.22 | -1.17 | -0.49 | Marotta et al. 2014 |
| Amazon 8 | 2°26'S | 54°54'W | 13.7 | 3.7 | µg C L^-1 day^-1 | 1.31 | 6.22 | -0.64 | -0.52 | Marotta et al. 2014 |
| Amazon 8 | 2°26'S | 54°54'W | 19.3 | 3.7 | µg C L^-1 day^-1 | 1.30 | 6.22 | 0.14 | -0.53 | Marotta et al. 2014 |
| Amazon 8 | 2°26'S | 54°54'W | 29.1 | 17.0 | µg C L^-1 day^-1 | 2.83 | 6.22 | 1.42 | 1.00 | Marotta et al. 2014 |
| Amazon 8 | 2°26'S | 54°54'W | 40.5 | 90.7 | µg C L^-1 day^-1 | 4.51 | 6.22 | 2.82 | 2.68 | Marotta et al. 2014 |
| Amazon 9 | 2°30'S | 54°57'W | 10.0 | 2.2 | µg C L^-1 day^-1 | 0.79 | 27.73 | -1.17 | -2.53 | Marotta et al. 2014 |
| Amazon 9 | 2°30'S | 54°57'W | 13.7 | 9.5 | µg C L^-1 day^-1 | 2.25 | 27.73 | -0.64 | -1.07 | Marotta et al. 2014 |
| Amazon 9 | 2°30'S | 54°57'W | 19.3 | 148.0 | µg C L^-1 day^-1 | 5.00 | 27.73 | 0.14 | 1.67 | Marotta et al. 2014 |
| Amazon 9 | 2°30'S | 54°57'W | 29.1 | 324.1 | µg C L^-1 day^-1 | 5.78 | 27.73 | 1.42 | 2.46 | Marotta et al. 2014 |
| Amazon 9 | 2°30'S | 54°57'W | 40.5 | 457.2 | µg C L^-1 day^-1 | 6.13 | 27.73 | 2.82 | 2.80 | Marotta et al. 2014 |
| Boreal 1 | 60°20'N | 17°45'E | 13.7 | 0.8 | µg C L^-1 day^-1 | -0.27 | 2.56 | -0.64 | -1.21 | Marotta et al. 2014 |
| Boreal 1 | 60°20'N | 17°45'E | 19.3 | 4.5 | µg C L^-1 day^-1 | 1.50 | 2.56 | 0.14 | 0.56 | Marotta et al. 2014 |
| Boreal 1 | 60°20'N | 17°45'E | 29.1 | 19.7 | µg C L^-1 day^-1 | 2.98 | 2.56 | 1.42 | 2.04 | Marotta et al. 2014 |
| Boreal 1 | 60°20'N | 17°45'E | 40.5 | 62.5 | µg C L^-1 day^-1 | 4.14 | 2.56 | 2.82 | 3.19 | Marotta et al. 2014 |
| Boreal 2 | 59°53'N | 17°57'E | 4.3 | 3.1 | µg C L^-1 day^-1 | 1.13 | 60.46 | -2.01 | -2.97 | Marotta et al. 2014 |
| Boreal 2 | 59°53'N | 17°57'E | 10.0 | 28.2 | µg C L^-1 day^-1 | 3.34 | 60.46 | -1.17 | -0.76 | Marotta et al. 2014 |
| Boreal 2 | 59°53'N | 17°57'E | 13.7 | 42.5 | µg C L^-1 day^-1 | 3.75 | 60.46 | -0.64 | -0.35 | Marotta et al. 2014 |
| Boreal 2 | 59°53'N | 17°57'E | 19.3 | 68.1 | µg C L^-1 day^-1 | 4.22 | 60.46 | 0.14 | 0.12 | Marotta et al. 2014 |
| Boreal 2 | 59°53'N | 17°57'E | 29.1 | 323.9 | µg C L^-1 day^-1 | 5.78 | 60.46 | 1.42 | 1.68 | Marotta et al. 2014 |
| Boreal 2 | 59°53'N | 17°57'E | 40.5 | 1114.8 | µg C L^-1 day^-1 | 7.02 | 60.46 | 2.82 | 2.91 | Marotta et al. 2014 |
| Boreal 3 | 59°52'N | 17°56'E | 4.3 | 7.3 | µg C L^-1 day^-1 | 1.99 | 91.67 | -2.01 | -2.53 | Marotta et al. 2014 |
| Boreal 3 | 59°52'N | 17°56'E | 10.0 | 18.7 | µg C L^-1 day^-1 | 2.93 | 91.67 | -1.17 | -1.59 | Marotta et al. 2014 |
| Boreal 3 | 59°52'N | 17°56'E | 13.7 | 85.2 | µg C L^-1 day^-1 | 4.44 | 91.67 | -0.64 | -0.07 | Marotta et al. 2014 |
| Boreal 3 | 59°52'N | 17°56'E | 19.3 | 132.3 | µg C L^-1 day^-1 | 4.89 | 91.67 | 0.14 | 0.37 | Marotta et al. 2014 |
| Boreal 3 | 59°52'N | 17°56'E | 29.1 | 571.4 | µg C L^-1 day^-1 | 6.35 | 91.67 | 1.42 | 1.83 | Marotta et al. 2014 |
| Boreal 3 | 59°52'N | 17°56'E | 40.5 | 1227.4 | µg C L^-1 day^-1 | 7.11 | 91.67 | 2.82 | 2.59 | Marotta et al. 2014 |
| Boreal 4 | 59°52'N | 17°10'E | 4.3 | 5.4 | µg C L^-1 day^-1 | 1.68 | 25.84 | -2.01 | -1.57 | Marotta et al. 2014 |
| Boreal 4 | 59°52'N | 17°10'E | 10.0 | 8.6 | µg C L^-1 day^-1 | 2.15 | 25.84 | -1.17 | -1.10 | Marotta et al. 2014 |
| Boreal 4 | 59°52'N | 17°10'E | 13.7 | 6.2 | µg C L^-1 day^-1 | 1.83 | 25.84 | -0.64 | -1.42 | Marotta et al. 2014 |
| Boreal 4 | 59°52'N | 17°10'E | 19.3 | 19.6 | µg C L^-1 day^-1 | 2.97 | 25.84 | 0.14 | -0.28 | Marotta et al. 2014 |
| Boreal 4 | 59°52'N | 17°10'E | 29.1 | 104.1 | µg C L^-1 day^-1 | 4.65 | 25.84 | 1.42 | 1.39 | Marotta et al. 2014 |
| Boreal 4 | 59°52'N | 17°10'E | 40.5 | 939.4 | µg C L^-1 day^-1 | 6.85 | 25.84 | 2.82 | 3.59 | Marotta et al. 2014 |
| Boreal 5 | 59°52'N | 15°11'E | 4.3 | 4.8 | µg C L^-1 day^-1 | 1.56 | 9.56 | -2.01 | -0.70 | Marotta et al. 2014 |
| Boreal 5 | 59°52'N | 15°11'E | 10.0 | 6.6 | µg C L^-1 day^-1 | 1.89 | 9.56 | -1.17 | -0.37 | Marotta et al. 2014 |
| Boreal 5 | 59°52'N | 15°11'E | 13.7 | 4.3 | µg C L^-1 day^-1 | 1.45 | 9.56 | -0.64 | -0.81 | Marotta et al. 2014 |
| Boreal 5 | 59°52'N | 15°11'E | 19.3 | 6.1 | µg C L^-1 day^-1 | 1.80 | 9.56 | 0.14 | -0.45 | Marotta et al. 2014 |
| Boreal 5 | 59°52'N | 15°11'E | 29.1 | 48.7 | µg C L^-1 day^-1 | 3.89 | 9.56 | 1.42 | 1.63 | Marotta et al. 2014 |
| Boreal 5 | 59°52'N | 15°11'E | 40.5 | 25.0 | µg C L^-1 day^-1 | 3.22 | 9.56 | 2.82 | 0.96 | Marotta et al. 2014 |
| Boreal 6 | 59°53'N | 15°15'E | 10.0 | 0.7 | µg C L^-1 day^-1 | -0.40 | 2.64 | -1.17 | -1.37 | Marotta et al. 2014 |
| Boreal 6 | 59°53'N | 15°15'E | 13.7 | 1.1 | µg C L^-1 day^-1 | 0.07 | 2.64 | -0.64 | -0.91 | Marotta et al. 2014 |
| Boreal 6 | 59°53'N | 15°15'E | 19.3 | 4.2 | µg C L^-1 day^-1 | 1.44 | 2.64 | 0.14 | 0.47 | Marotta et al. 2014 |
| Boreal 6 | 59°53'N | 15°15'E | 29.1 | 30.9 | µg C L^-1 day^-1 | 3.43 | 2.64 | 1.42 | 2.46 | Marotta et al. 2014 |
| Boreal 6 | 59°53'N | 15°15'E | 40.5 | 12.8 | µg C L^-1 day^-1 | 2.55 | 2.64 | 2.82 | 1.58 | Marotta et al. 2014 |
| Boreal 7 | 59°54'N | 15°23'E | 10.0 | 3.7 | µg C L^-1 day^-1 | 1.30 | 10.47 | -1.17 | -1.05 | Marotta et al. 2014 |
| Boreal 7 | 59°54'N | 15°23'E | 13.7 | 4.2 | µg C L^-1 day^-1 | 1.44 | 10.47 | -0.64 | -0.91 | Marotta et al. 2014 |
| Boreal 7 | 59°54'N | 15°23'E | 19.3 | 13.6 | µg C L^-1 day^-1 | 2.61 | 10.47 | 0.14 | 0.26 | Marotta et al. 2014 |
| Boreal 7 | 59°54'N | 15°23'E | 29.1 | 66.0 | µg C L^-1 day^-1 | 4.19 | 10.47 | 1.42 | 1.84 | Marotta et al. 2014 |
| Boreal 7 | 59°54'N | 15°23'E | 40.5 | 91.1 | µg C L^-1 day^-1 | 4.51 | 10.47 | 2.82 | 2.16 | Marotta et al. 2014 |
| Boreal 8 | 59°56'N | 15°24'E | 4.3 | 3.7 | µg C L^-1 day^-1 | 1.31 | 4.88 | -2.01 | -0.28 | Marotta et al. 2014 |
| Boreal 8 | 59°56'N | 15°24'E | 10.0 | 2.5 | µg C L^-1 day^-1 | 0.90 | 4.88 | -1.17 | -0.68 | Marotta et al. 2014 |
| Boreal 8 | 59°56'N | 15°24'E | 13.7 | 2.7 | µg C L^-1 day^-1 | 1.00 | 4.88 | -0.64 | -0.58 | Marotta et al. 2014 |
| Boreal 8 | 59°56'N | 15°24'E | 19.3 | 1.7 | µg C L^-1 day^-1 | 0.51 | 4.88 | 0.14 | -1.08 | Marotta et al. 2014 |
| Boreal 8 | 59°56'N | 15°24'E | 29.1 | 19.7 | µg C L^-1 day^-1 | 2.98 | 4.88 | 1.42 | 1.39 | Marotta et al. 2014 |
| Boreal 8 | 59°56'N | 15°24'E | 40.5 | 21.9 | µg C L^-1 day^-1 | 3.08 | 4.88 | 2.82 | 1.50 | Marotta et al. 2014 |
| Stechlin 15-20 | 53°8′N | 13°1′E | 4.0 | 1.4 | nmol CH4 g_dw^-1 h^-1 | 0.35 | 4.82 | -2.05 | -1.22 | Fuchs et al. 2016 |
| Stechlin 15-20 | 53°8′N | 13°1′E | 8.0 | 3.3 | nmol CH4 g_dw^-1 h^-1 | 1.18 | 4.82 | -1.46 | -0.39 | Fuchs et al. 2016 |
| Stechlin 15-20 | 53°8′N | 13°1′E | 12.0 | 2.6 | nmol CH4 g_dw^-1 h^-1 | 0.94 | 4.82 | -0.88 | -0.63 | Fuchs et al. 2016 |
| Stechlin 20-25 | 53°8′N | 13°1′E | 4.0 | 2.2 | nmol CH4 g_dw^-1 h^-1 | 0.80 | 4.71 | -2.05 | -0.75 | Fuchs et al. 2016 |
| Stechlin 20-25 | 53°8′N | 13°1′E | 8.0 | 2.3 | nmol CH4 g_dw^-1 h^-1 | 0.85 | 4.71 | -1.46 | -0.70 | Fuchs et al. 2016 |
| Stechlin 20-25 | 53°8′N | 13°1′E | 12.0 | 3.5 | nmol CH4 g_dw^-1 h^-1 | 1.26 | 4.71 | -0.88 | -0.28 | Fuchs et al. 2016 |
| Geneva 15-20 | 46°24′N | 6°25′E | 4.0 | 1.8 | nmol CH4 g_dw^-1 h^-1 | 0.60 | 2.94 | -2.05 | -0.48 | Fuchs et al. 2016 |
| Geneva 15-20 | 46°24′N | 6°25′E | 8.0 | 2.7 | nmol CH4 g_dw^-1 h^-1 | 1.00 | 2.94 | -1.46 | -0.08 | Fuchs et al. 2016 |
| Geneva 15-20 | 46°24′N | 6°25′E | 12.0 | 2.3 | nmol CH4 g_dw^-1 h^-1 | 0.82 | 2.94 | -0.88 | -0.26 | Fuchs et al. 2016 |
| Geneva 20-25 | 46°24′N | 6°25′E | 4.0 | 0.5 | nmol CH4 g_dw^-1 h^-1 | -0.80 | 9.02 | -2.05 | -3.00 | Fuchs et al. 2016 |
| Geneva 20-25 | 46°24′N | 6°25′E | 8.0 | 2.0 | nmol CH4 g_dw^-1 h^-1 | 0.67 | 9.02 | -1.46 | -1.53 | Fuchs et al. 2016 |
| Geneva 20-25 | 46°24′N | 6°25′E | 12.0 | 2.2 | nmol CH4 g_dw^-1 h^-1 | 0.78 | 9.02 | -0.88 | -1.42 | Fuchs et al. 2016 |

**Supplementary Table 2 |** **Summary of lake sediment methane production rates by the end of the 21^st^ century in 57 globally distributed lakes**. Shown as the names of each lake, their location (longitude and latitude), surface area (km^2^), mean depth (m), and simulated CH_4_ production rates by the end of the 21^st^ century, under RCPs 2.6–8.5, relative to the historical global average.

| **Lake** | **Longitude** | **Latitude** | **Area (km^2^)** | **Mean depth (m)** | **RCP 2.6** | **RCP 6.0** | **RCP 8.5** |
| --- | --- | --- | --- | --- | --- | --- | --- |
| Allequash Lake | -89.75 | 46.25 | 1.64 | 2.9 | 1.59 | 1.82 | 2.13 |
| Annecy | 6.25 | 45.75 | 27 | 41 | 1.03 | 0.96 | 1.09 |
| Annie | -81.25 | 27.25 | 0.36 | - | 7.17 | 7.71 | 8.62 |
| Argyle | 128.75 | -16.25 | 980 | 10.1 | 10.27 | 12.19 | 13.89 |
| Biel | 7.25 | 47.25 | 39.3 | 30 | 0.89 | 0.96 | 1.08 |
| Big Muskellunge Lake | -89.75 | 46.25 | 3.63 | 7.5 | 0.79 | 0.81 | 0.84 |
| Black Oak Lake | -89.25 | 46.25 | 2.28 | 10.36 | 0.98 | 1.05 | 1.18 |
| Bourget | 5.75 | 45.75 | 44 | 80 | 0.88 | 0.96 | 1.08 |
| Burley Griffin | 149.25 | -35.25 | - | - | 2.95 | 3.08 | 3.60 |
| Crystal Bog | -89.75 | 46.25 | 0.01 | 1.7 | 3.12 | 3.85 | 4.91 |
| Crystal Lake | -89.75 | 46.25 | 0.38 | 10.4 | 0.91 | 0.94 | 0.98 |
| Delavan | -88.75 | 42.75 | 6.96 | 7.61 | 1.70 | 1.74 | 2.05 |
| Dickie Lake | -79.25 | 45.25 | 0.94 | 5 | 0.73 | 0.78 | 0.82 |
| Eagle Lake | -76.75 | 44.75 | 6.65 | 10.1 | 0.82 | 0.85 | 0.86 |
| Ekoln basin of Malaren | 17.75 | 59.75 | 20.18 | 11.5 | 0.82 | 0.91 | 1.03 |
| Erken | 18.75 | 59.75 | 24 | 9 | 0.87 | 0.96 | 1.08 |
| Esthwaite Water | -2.75 | 54.25 | 0.96 | 6.9 | 0.81 | 0.86 | 0.91 |
| Falling Creek Reservoir | -79.75 | 37.25 | 119 | 4 | 4.72 | 5.74 | 6.80 |
| Feeagh | -9.75 | 53.75 | 3.9 | 14.5 | 1.06 | 1.12 | 1.19 |
| Fish Lake | -89.75 | 43.25 | 0.8 | 6.6 | 0.91 | 0.94 | 1.07 |
| Geneva | 6.75 | 46.25 | 580.1 | 152.7 | 0.77 | 0.76 | 0.77 |
| Great Pond | -69.75 | 44.75 | - | - | 0.96 | 1.05 | 1.16 |
| Green Lake | -88.75 | 43.75 | 29.48 | 33.55 | 0.65 | 0.66 | 0.72 |
| Harp Lake | -79.25 | 45.25 | 0.71 | 13.32 | 0.62 | 0.62 | 0.63 |
| Kilpisjarvi | 20.75 | 69.25 | 37.3 | 20 | 0.60 | 0.61 | 0.61 |
| Kinneret | 35.75 | 32.75 | 168 | 24 | 6.06 | 6.54 | 7.41 |
| Kuivajarvi | 24.25 | 61.75 | 0.62 | 6.3 | 1.67 | 1.96 | 2.19 |
| Langtjern | 9.75 | 60.25 | 0.23 | 2 | 0.63 | 0.63 | 0.64 |
| Laramie Lake | -105.75 | 40.75 | 0.14 | - | 0.79 | 0.90 | 1.03 |
| Lower Zurich | 8.75 | 47.25 | 67 | 49 | 0.76 | 0.82 | 0.93 |
| Mendota | -89.25 | 43.25 | 39.61 | 12.8 | 1.19 | 1.23 | 1.38 |
| Mozhaysk | 35.75 | 55.75 | 30.7 | 7 | 0.64 | 0.68 | 0.72 |
| Mt Bold | 138.75 | -35.25 | 3.08 | 13 | 2.47 | 2.63 | 2.84 |
| Mueggelsee | 13.75 | 52.25 | 7.4 | 4.9 | 3.89 | 4.48 | 5.43 |
| Neuchatel | 6.75 | 46.75 | 217 | 64 | 0.70 | 0.74 | 0.82 |
| Nohipalo Mustjarv | 27.25 | 57.75 | 0.22 | 3.9 | 2.66 | 3.11 | 3.63 |
| Nohipalo Valgejarv | 27.25 | 57.75 | 0.07 | 6.2 | 2.04 | 2.46 | 2.75 |
| Okauchee Lake | -88.25 | 43.25 | 4.9 | 7.62 | 0.69 | 0.70 | 0.74 |
| Rappbode Reservoir | 10.75 | 51.75 | 3.95 | 28.6 | 0.65 | 0.69 | 0.76 |
| Rimov | 14.25 | 48.75 | 2.11 | 16 | 0.66 | 0.69 | 0.72 |
| Rotorua | 176.25 | -38.25 | 425 | 10.8 | 3.52 | 3.97 | 4.54 |
| Sammamish | -122.25 | 47.75 | 19.8 | 17.7 | 1.19 | 1.33 | 1.57 |
| Sau Reservoir | 2.25 | 41.75 | 5.8 | 29 | 1.93 | 2.05 | 2.31 |
| Sparkling Lake | -89.75 | 46.25 | 0.64 | 10.9 | 0.74 | 0.76 | 0.79 |
| Stechlin | 13.25 | 53.25 | 2.23 | 23.2 | 0.65 | 0.69 | 0.76 |
| Sunapee | -72.25 | 43.25 | 16.55 | 11.4 | 0.79 | 0.82 | 0.84 |
| Tahoe | -120.25 | 39.25 | 490 | 304 | 0.74 | 0.74 | 0.73 |
| Tarawera | 176.25 | -38.25 | 41.3 | 50 | 2.05 | 2.29 | 2.56 |
| Toolik Lake | -149.75 | 68.75 | - | - | 0.58 | 0.59 | 0.60 |
| Trout Bog | -89.75 | 46.25 | 0.001 | 5.6 | 0.62 | 0.64 | 0.65 |
| Trout Lake | -89.75 | 46.25 | 15.65 | 14.6 | 0.67 | 0.67 | 0.69 |
| Two Sisters Lake | -89.75 | 45.75 | 2.91 | 9.14 | 1.27 | 1.36 | 1.48 |
| Vendyurskoe | 33.25 | 62.25 | 10..4 | 5.3 | 0.69 | 0.77 | 0.87 |
| Vortsjarv | 26.25 | 58.25 | 270 | 2.8 | 2.67 | 3.05 | 3.67 |
| Washington | -122.25 | 47.75 | 87.6 | 33 | 1.22 | 1.26 | 1.43 |
| Windermere | -2.75 | 54.25 | 14.76 | 21.3 | 0.73 | 0.78 | 0.86 |
| Wingra | -89.25 | 43.25 | 1.36 | 2.7 | 3.86 | 4.55 | 5.50 |

**Supplementary Table 3 |** **Comparison of empirical and modeled methanogenesis rates.** Mean annual methanogenesis rate for each of the 17 sites in Marotta et al. (2014) estimated with Eq. 1, using modelled daily historical (1970–1999) lake bottom temperatures at each site’s corresponding 0.5°-by-0.5° grid and setting *E*_a_' = 0.96 eV and *T*_c_ to the local annual mean lake bottom temperature rather than the global mean. *J*(*T*_c_) was estimated for each site via log-linear regression of the rate versus temperature. Daily rates (*J*(*T*)) were averaged to obtain the annual historical mean rate for each site. The rate ratio of tropical and boreal site means was then compared to the ratio of modelled relative historical methanogenesis rates (Fig. 1b) at the same grid cells. 95% confidence intervals of the ratios of the mean rates were computed following the method of Fieller (1954), using bootstrapped estimates of the standard deviation.

|  | **Simulation-based** | | **Incubation-based** | |
| --- | --- | --- | --- | --- |
| **Site** | **Annual mean historical bottom temperature (°C)** | **Historical methanogenesis rate relative to global mean (-)** | ***J*(*T*_c_)**  **(µg C L^−1^ day^−1^)** | **Annual mean methanogenesis rate**  **(µg C L^−1^ day^−1^)** |
| Amazon 1 | 29,87 | 13,30 | 51,70 | 51,75 |
| Amazon 2 | 32,22 | 17,67 | 9,16 | 9,18 |
| Amazon 3 | 32,22 | 17,67 | 62,84 | 62,99 |
| Amazon 4 | 30,78 | 14,85 | 11,73 | 11,75 |
| Amazon 5 | 30,78 | 14,85 | 21,89 | 21,92 |
| Amazon 6 | 30,78 | 14,85 | 129,77 | 129,95 |
| Amazon 7 | 30,78 | 14,85 | 36,22 | 36,27 |
| Amazon 8 | 29,87 | 13,30 | 22,14 | 22,16 |
| Amazon 9 | 29,87 | 13,30 | 199,49 | 199,67 |
| mean | 30,80 | 14,96 | 60,55 | 60,63 |
| sd | 0,91 | 1,55 | 57,33 | 57,51 |
|  |  |  |  |  |
| Boreal 1 | 5,89 | 0,76 | 0,29 | 0,39 |
| Boreal 2 | 6,38 | 0,90 | 9,00 | 13,04 |
| Boreal 3 | 6,38 | 0,90 | 14,80 | 21,45 |
| Boreal 4 | 6,24 | 0,84 | 3,88 | 5,33 |
| Boreal 5 | 5,71 | 0,70 | 4,16 | 5,16 |
| Boreal 6 | 5,71 | 0,70 | 0,56 | 0,69 |
| Boreal 7 | 5,71 | 0,70 | 2,08 | 2,58 |
| Boreal 8 | 5,71 | 0,70 | 2,01 | 2,49 |
| mean | 5,97 | 0,78 | 4,60 | 6,39 |
| sd | 0,29 | 0,09 | 4,41 | 6,48 |
|  |  |  |  |  |
| ratio |  | 19,34 |  | 9,49 |
| Lower 95% CI |  | 17,34 |  | 2,75 |
| Upper 95% CI |  | 21,83 |  | 41,74 |

**Supplementary References**

Duc, N. T., P. Crill, and D. Bastviken. 2010. Implications of temperature and sediment characteristics on methane formation and oxidation in lake sediments. Biogeochemistry **100**: 185–196. doi:10.1007/s10533-010-9415-8

Fieller, E. C. 1954. Some Problems in Interval Estimation. J. R. Stat. Soc. Ser. B **16**: 175–185. doi:10.1111/j.2517-6161.1954.tb00159.x

Fuchs, A., E. Lyautey, B. Montuelle, and P. Casper. 2016. Effects of increasing temperatures on methane concentrations and methanogenesis during experimental incubation of sediments from oligotrophic and mesotrophic lakes. J. Geophys. Res. Biogeosciences **121**: 1394–1406. doi:10.1002/2016JG003328

Kourzeneva, E. 2010. External data for lake parameterization in Numerical Weather Prediction and climate modeling. Boreal Environ. Res. **15**: 165–177.

Liikanen, A., T. Murtoniemi, H. Tanskanen, T. Vaisanen, and P. J. Martikainen. 2002. Effects of Temperature and Oxygen Availability on Greenhouse Gas and Nutrient Dynamics in Sediment of a Eutrophic Mid-Boreal Lake. Biogeochemistry **59**: 269–286. doi:10.1023/A:1016015526712

Maberly, S. C., R. A. O’Donnell, R. I. Woolway, and others. 2020. Global lake thermal regions shift under climate change. Nat. Commun. **11**: 1232. doi:10.1038/s41467-020-15108-z

Marotta, H., L. Pinho, C. Gudasz, D. Bastviken, L. J. Tranvik, and A. Enrich-Prast. 2014. Greenhouse gas production in low-latitude lake sediments responds strongly to warming. Nat. Clim. Chang. **4**: 467–470. doi:10.1038/nclimate2222

Mesman, J. P., A. I. Ayala, R. Adrian, and others. 2020. Performance of one-dimensional hydrodynamic lake models during short-term extreme weather events. Environ. Model. Softw. **133**: 104852. doi:10.1016/j.envsoft.2020.104852

Pilla, R. M., C. E. Williamson, B. V. Adamovich, and others. 2020. Deeper waters are changing less consistently than surface waters in a global analysis of 102 lakes. Sci. Rep. **10**: 20514. doi:10.1038/s41598-020-76873-x

Schulz, S., H. Matsuyama, and R. Conrad. 1997. Temperature dependence of methane production from different precursors in a profundal sediment (Lake Constance). FEMS Microbiol. Ecol. **22**: 207–213. doi:10.1111/j.1574-6941.1997.tb00372.x

Shatwell, T., W. Thiery, and G. Kirillin. 2019. Future projections of temperature and mixing regime of European temperate lakes. Hydrol. Earth Syst. Sci. **23**: 1533–1551. doi:10.5194/hess-23-1533-2019

Woolway, R. I., E. Jennings, T. Shatwell, M. Golub, D. C. Pierson, and S. C. Maberly. 2021a. Lake heatwaves under climate change. Nature **589**. doi:10.1038/s41586-020-03119-1

Woolway, R. I., and C. J. Merchant. 2018. Intralake Heterogeneity of Thermal Responses to Climate Change: A Study of Large Northern Hemisphere Lakes. J. Geophys. Res. Atmos. **123**: 3087–3098. doi:10.1002/2017JD027661

Woolway, R. I., and C. J. Merchant. 2019. Worldwide alteration of lake mixing regimes in response to climate change. Nat. Geosci. **12**: 271–276. doi:10.1038/s41561-019-0322-x

Woolway, R. I., S. Sharma, G. A. Weyhenmeyer, and others. 2021b. Phenological shifts in lake stratification under climate change. Nat. Commun. **12**: 1–11. doi:10.1038/s41467-021-22657-4

Yvon-Durocher, G., A. P. Allen, D. Bastviken, R. Conrad, C. Gudasz, A. St-Pierre, N. Thanh-Duc, and P. A. Del Giorgio. 2014. Methane fluxes show consistent temperature dependence across microbial to ecosystem scales. Nature **507**: 488–491. doi:10.1038/nature13164

Zeikus, J. G., and M. R. Winfrey. 1976. Temperature limitation of methanogenesis in aquatic sediments. Appl. Environ. Microbiol. **31**: 99–107.
